# The role of the microbiota and metabolites in the treatment of pulmonary fibrosis with UC-MSCs: Integrating fecal metabolomics and 16S rDNA analysis

**DOI:** 10.1371/journal.pone.0313989

**Published:** 2025-01-09

**Authors:** Yukai Luo, Shuang Zhou, Xiaojing Zhang, Yijian Lin, Jun Liu, Wenzhao Cheng, Yiming Zeng

**Affiliations:** 1 Fujian Key Laboratory of Lung Stem Cells, The Second Affiliated Hospital of Fujian Medical University, Quanzhou, Fujian, China; 2 The Second Clinical Medical School of Fujian Medical University, Quanzhou, Fujian, China; 3 Department of Respiratory and Critical Care Medicine, The Second Hospital of Longyan, Longyan, Fujian, China; 4 Jinan Microecological Biomedicine Shandong Laboratory, Shounuo City Light West Block, Jinan, Shandong, China; 5 Respiratory Medicine Center of Fujian Province, The Second Affiliated Hospital of Fujian Medical University, Quanzhou, Fujian, China; South China Agricultural University, CHINA

## Abstract

**Introduction:**

Pulmonary fibrosis (PF) is a chronic and irreversible interstitial lung disease characterized by a lack of effective therapies. Mesenchymal stem cells (MSCs) have garnered significant interest in the realm of lung regeneration due to their abundant availability, ease of isolation, and capacity for expansion. The objective of our study was to investigate the potential therapeutic role of umbilical cord-derived MSCs (UC-MSCs) in the management of PF, with a focus on the alterations in the gut microbiota and its metabolites during the use of UC-MSCs for the treatment of pulmonary fibrosis, as well as the possible mechanisms involved.

**Methods:**

Bleomycin injection was utilized to establish a mouse model of lung fibrosis, followed by the application of 16S rDNA sequencing and LC–MS/MS metabolomics to explore the underlying mechanism of UC-MSC treatment for lung fibrosis. Seventy-five mice were allocated into five groups, namely Control, Model, and low/medium/high dose of UC-MSCs groups, and survival metrics, lung morphology, and the levels of the inflammatory cytokines TNF-α, IL-1β, IL-6, and TGF-β1 were subsequently evaluated. Fecal samples from six mice in each of the Control group, Model group, and UC-MSCs-M groups were collected randomly for 16S rDNA sequencing to analyze the gut microbiota and nontargeted metabolomics.

**Results:**

In comparison to IPF model mice, the three treatment groups exhibited increased survival rates, restored alveolar morphology, and reduced levels of the inflammatory cytokines TNF-α, IL-1β, IL-6, and TGF-β1, confirming the anti-inflammatory properties of UC-MSCs in IPF treatment. The findings from the 16S rDNA assay indicate that UC-MSCs treatment effectively lower α-diversity induced such as Chao 1 and ACE, as well as β-diversity, leading to a decrease in microbiota abundance. The findings from the metabolomics analysis revealed that the metabolites exhibiting notable variances were primarily composed of Lipids and lipid-like molecules, Organoheterocyclic compounds, Organic acids and derivatives, and Benzenoids, indicating the potential of UC-MSCs to exert antifibrotic effects via these metabolic pathways.

**Conclusion:**

Umbilical cord-derived mesenchymal stem cells (UC-MSCs) ameliorate bleomycin-induced pulmonary fibrosis symptoms in mice by exerting anti-inflammatory effects and mitigating pulmonary fibrosis through the modulation of gut microbiota disorders and their metabolism. These findings offer novel insights into the potential mechanisms and clinical utility of stem cell therapy for pulmonary fibrosis.

## 1. Introduction

Pulmonary fibrosis (PF) is a debilitating respiratory condition characterized by progressive lung damage and scarring. Idiopathic pulmonary fibrosis (IPF) is the most prevalent form of PF, and is characterized by irreversible destruction of alveoli, widespread restructuring of lung tissue, and accumulation of the extracellular matrix. The pathogenesis of pulmonary fibrosis involves various factors, including inflammation, epithelial–mesenchymal transition (EMT), and oxidative stress. While pirfenidone and nintedanib have been authorized for IPF treatment, their efficacy has not been significantly impactful. Currently, lung transplantation remains the only viable treatment option for this condition [1–4]. Consequently, there is a pressing need to explore and develop alternative therapies.

Mesenchymal stem cells (MSCs) represent a distinct cellular population characterized by diverse differentiation and self-renewal capacities, and are derived from various tissues including bone marrow, umbilical cord, and adipose tissue [5]. Notably, umbilical cord-derived MSCs (UC-MSCs) exhibit superior expansion and differentiation potential compared with bone marrow-derived MSCs (BM-MSCs) [6]. Existing research has established a correlation between the progression of IPF and the inflammatory response [7, 8]. MSCs have been shown to repair cells and tissues by exerting anti-inflammatory effects and modulating immune cell activities [9]. Additional research has indicated that MSCs play a role in reducing fibrosis in lung fibrosis models by mitigating inflammatory responses and decreasing collagen deposition [10]. Nevertheless, the precise molecular mechanisms through which MSCs alleviate pulmonary fibrosis remain unclear.

The gut microbiota, which is the most extensive and populous microbial community within the human body, serves a crucial function in maintaining the intricate equilibrium of the host’s metabolic and immune functions [11]. Recent studies have revealed a correlation between dysbiosis of the gut microbiota and the advancement of IPF [12, 13]. The gut microbiota compartment forms the foundation of immunodynamics and governs the reaction of the lung to inflammation primarily by modulating innate immune cells, adjusting inflammatory cytokine reactions, and reshaping adaptive immunity, thereby potentially disrupting this delicate balance [14, 15]. An increasing body of research has revealed a correlation between lung disease and intestinal disease, leading to the conceptualization of the "gut–lung axis". While previous studies have demonstrated the impact of factors such as diet [16], on the progression of IPF through the modulation of this axis [12, 17], the specific mechanism by which UC-MSCs influence intestinal microbial organisms remains unreported. Thus, the aim of this study was to further investigate the relationship between gut microbial organisms and IPF.

Numerous histological and microbiological analyses have been widely used in the examination of the gut microbiota in diverse diseases [18, 19]. Nevertheless, the integration of the gut microbiota and metabolomic analysis in the context of IPF remains unexplored in the literature. Thus, the present study aimed to investigate the anti-inflammatory properties of UC-MSCs in a murine model of pulmonary fibrosis, while also evaluating their impact on the gut microbiota and metabolites. This research seeks to offer novel perspectives on therapeutic approaches for IPF.

## 2. Results

### 2.1 UC-MSCs administration attenuates symptoms in BLM-induced model IPF mice

**Fig 1:**
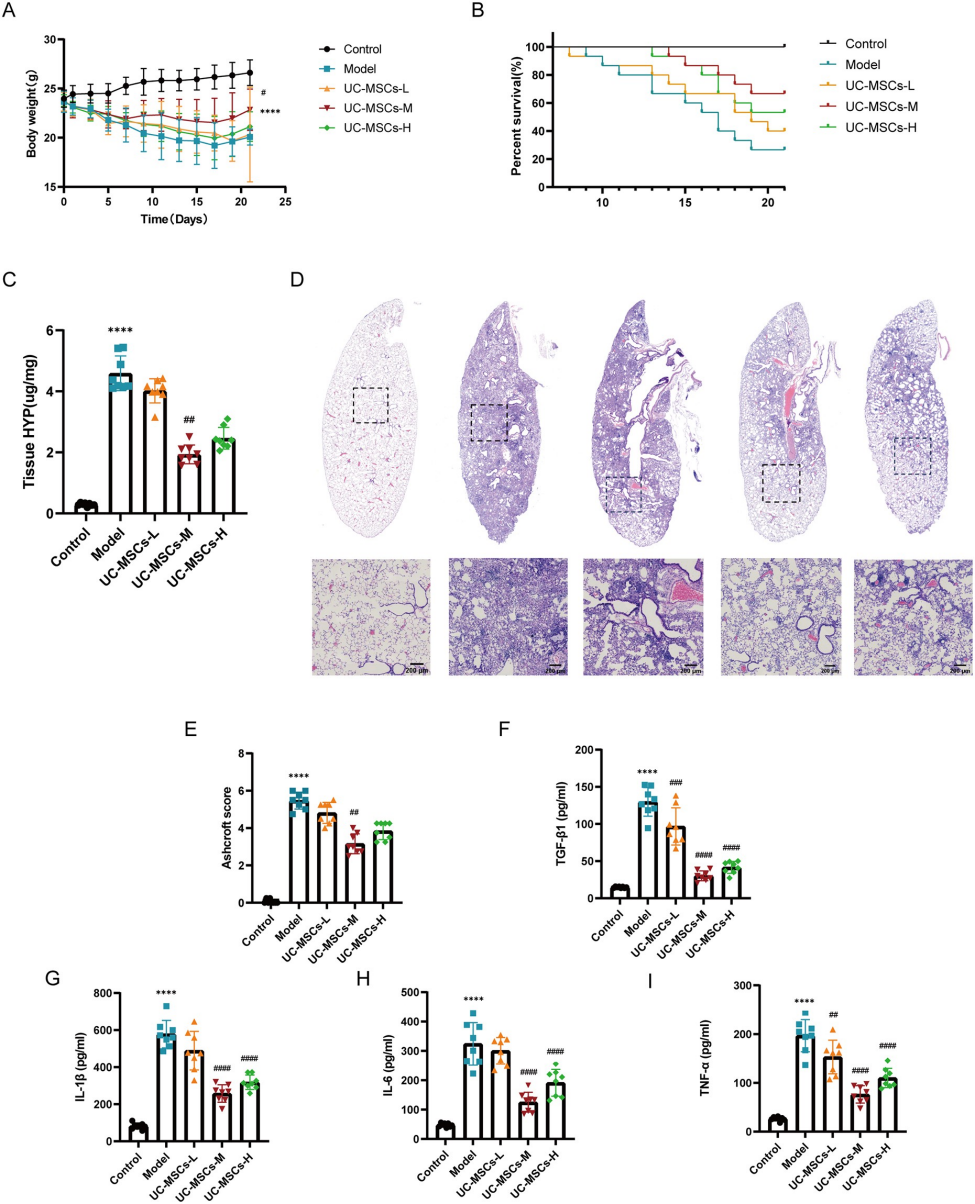
The effects of UC-MSCs on BLM-induced IPF severity and cytokine production. (**A**) Body weight. (**B**) Survival probability. (**C**) Examination of HYP content in lung tissues. (**D**) Representative lung histological morphology. (**E**) Ashcroft score. (**F-I**) The concentrations of TNF-ɑ, IL-1β, IL-6 and TGF-β1 in the broncho-alveolar lavage fluid (BALF) was evaluated by ELISA kits. The data are shown as the means ± SDs (Fig 1A, 1B, n = 15; Fig 1C-1I, n = 8); (* *p* < 0.05, **p< 0.01, *** *p* < 0.001, **** *p* < 0.0001 Model vs. Control; # *p* < 0.05, ## p < 0.01, ###p<0.001, ####p<0.0001 vs. Model).

On the seventh day following BLM administration, the average body weight of the mice in the BLM group was significantly lower than that of the control group (Fig 1A). On the ninth day after BLM administration, the weights of the mice in the UC-MSCs intervention groups began to increase, resulting in significantly greater average weights than those of the Model group at the end of the study period. Additionally, survival probability was utilized to evaluate the impact of UC-MSCs treatment on BLM-induced IPF model mice, with Kaplan–Meier survival analysis indicating that intravenous administration of UC-MSCs led to increased overall survival rates and reduced median survival times in mice with pulmonary fibrosis (Fig 1B). Collectively, these findings suggest that supplementation with UC-MSCs effectively mitigated the severity of symptoms associated with BLM- induced injury in mice. At the end of the experiment, the number of survival mice in control group, model group, UC-MSCs-L group, UC-MSCs-M group and UC-MSCs-H group was 15, 4, 7, 10 and 8, respectively. The assessment of hydroxyproline levels, a widely accepted indicator of pulmonary fibrosis severity, was conducted across the five experimental groups. Results showed a significant increase in hydroxyproline content in mice treated with BLM compared to the Control group, while administration of UC-MSCs led to a reduction in hydroxyproline levels in certain individuals (Fig 1C). Importantly, the increase in survival indicators did not exhibit a linear relationship with increasing doses of UC-MSCs, and the effectiveness of moderate-dose UC-MSCs surpassed that of both low- and high-dose UC-MSCs. These findings collectively suggest that supplementation with UC-MSCs effectively mitigates the severity of symptoms associated with mice with pulmonary fibrosis in mice.

### 2.2 Administration of UC-MSCs abrogates lung injury in BLM-induced model mice

To evaluate the effect of UC-MSCs on BLM-induced lung damage, morphological changes in the lungs of mice were examined. As observed in the HE-stained lung sections (Fig 1D), mice in the Control group had intact alveoli with transparent alveolar walls and no obvious inflammatory response. In contrast, tracheal instillation of BLM resulted in lung injury and inflammation, inflammatory cells and fibroblasts infiltrated the alveolar spaces, and the alveoli were disrupted, resulting in enlarged alveolar spaces and thickened alveolar walls. The administration of UC-MSCs effectively reversed BLM-induced pulmonary fibrosis in mice, as indicated by a decrease in the inflammatory cell count in lung tissues and the presence of a smaller area of injured tissue surrounded by larger areas with a typical alveolar structure (Fig 1D). Additionally, the Ashcroft score was significantly lower in lung tissues from the UC-MSCs-M and UC-MSCs-H groups, than in those from the Model group (Fig 1E). Furthermore, our findings suggest that compared with both the high-dose and low-dose treatment groups, the medium-dose treatment group exhibited greater alleviation of IPF. These results highlight the protective effects of UC-MSCs on the development of BLM-induced pulmonary fibrosis.

### 2.3 UC-MSCs suppresses inflammatory cytokine release in the BLM-induced model mice

The effect of UC-MSCs treatment on inflammatory activity was subsequently assessed through the quantification of cytokine levels in the lung tissues of the experimental mice, as depicted in Fig 1F–1I, compared with those in the Control group, the concentrations of the proinflammatory cytokines IL-1β, IL-6, TNF-α, and the pro-fibrotic factor TGF-β1 in the bronchoalveolar lavage fluid (BALF) of the Model group were markedly elevated.

The administration of UC-MSCs resulted in a decrease in cytokine levels compared with those found in the Model group. Furthermore, the reduction in inflammatory factors was notably more pronounced in the medium-dose treatment group than in to the low-dose and high-dose groups. These findings indicate that UC-MSCs have the potential to mitigate BLM-induced IPF through the inhibition of cytokine production.

### 2.4 UC-MSCs changed the profiles of GM in BLM-induced model mice

The GM is considered a major environmental factor that plays a significant role in the development of IPF and lung damage. In this study, we investigated the effect of MSCs on the GM by multiplex sequencing of 16S rDNA in BLM-induced IPF model mice.

**Fig 2:**
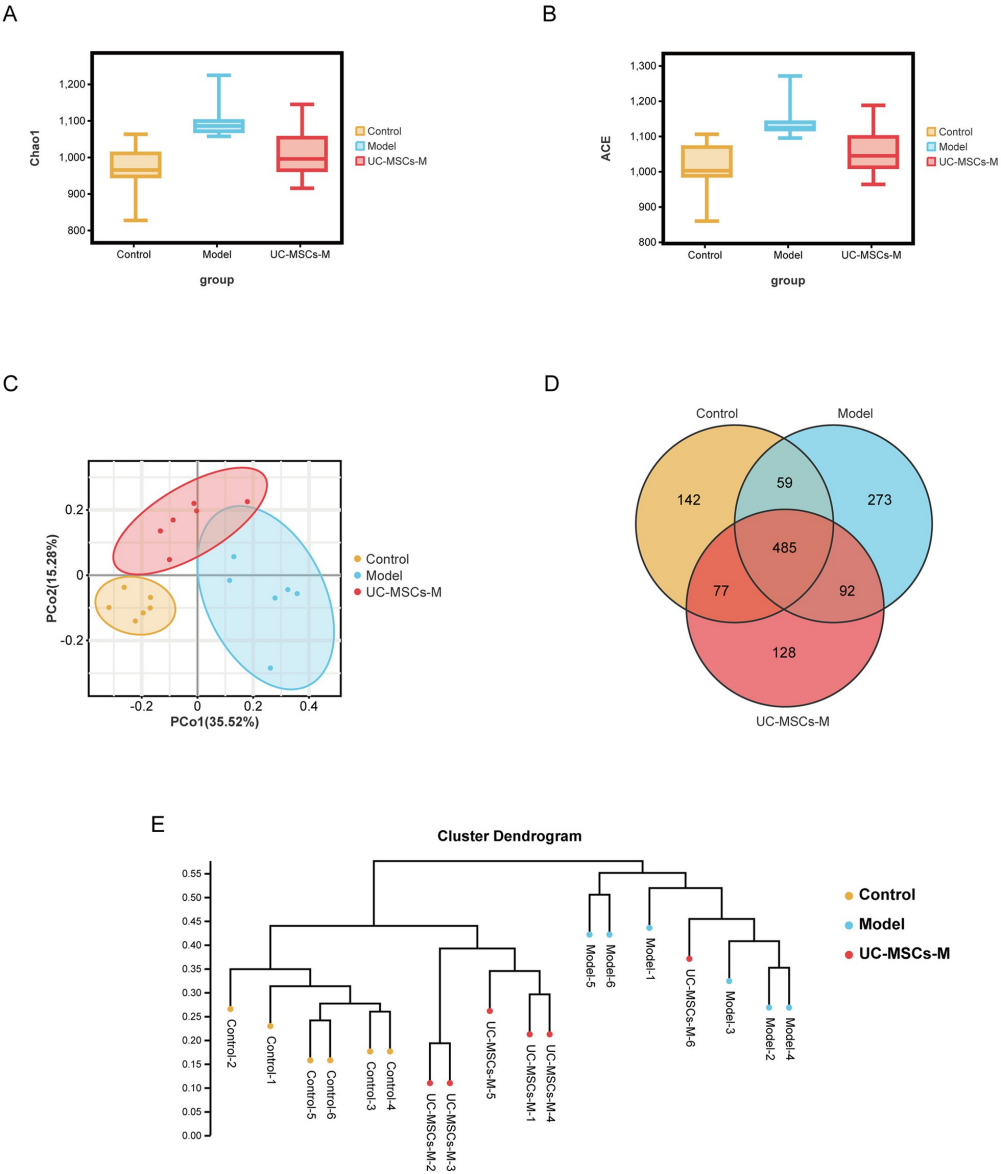
Mesenchymal stem cell therapy alters the gut microbiota (GM) composition in BLM-induced IPF model mice. (**A**) Chao1 richness index of the GM. (**B**) ACE richness index of the GM. (**C**) PCoA plot of microbial communities based on the OUT abundances. (**D**) Venn diagram showing the distribution of operational taxonomic units (OTUs) among different groups. (**E**) Clustering analysis of gut microbiota among samples based on the Bray method (OTU abundance). The data are shown as the means ± SDs (n = 6 for all the groups).

The Chao1 index tended to increase in BLM-injured mice in the Model group and were significantly decreased in the UC-MSCs-treated UC-MSCs-M group (Fig 2A). Moreover, BLM increased the ACE diversity index, whereas MSCs tended to decrease this index (Fig 2B). Additionally, to better compare the bacterial community similarities among the three groups, principal coordinate analysis (PCoA) was performed on the OTU abundances obtained from the three groups samples. As shown in Fig 2C, the PCoA plot indicated an apparent clustering in the microbial composition of each group. There was a significant difference in the microbial composition of the Model and Control groups, whereas there was a partial overlap between the microbial composition of the UC-MSCs-M group and the Model group. A Venn diagram revealed that all of the groups had unique and shared operational taxonomic units (OTUs) (Fig 2D). In the clustering analysis (Fig 2E), samples in the Model group were clustered far away from the Control group. On the contrary, both the samples in the Control and UC-MSCs-M groups were clustered within the same cluster, which further supported the restoration of the GM in the UC-MSCs-M group by treatment of the BLM-injured mice with MSCs. Collectively, these data indicate that the UC-MSCs treatment can alleviate the BLM-induced disturbances in the GM profile of mice.

### 2.5 Composition of the GM is modulated by UC-MSCs in BLM-induced model mice

The changes in the composition of the intestinal microbiome at the phylum and genus level in our experimental mice were significant.

**Fig 3:**
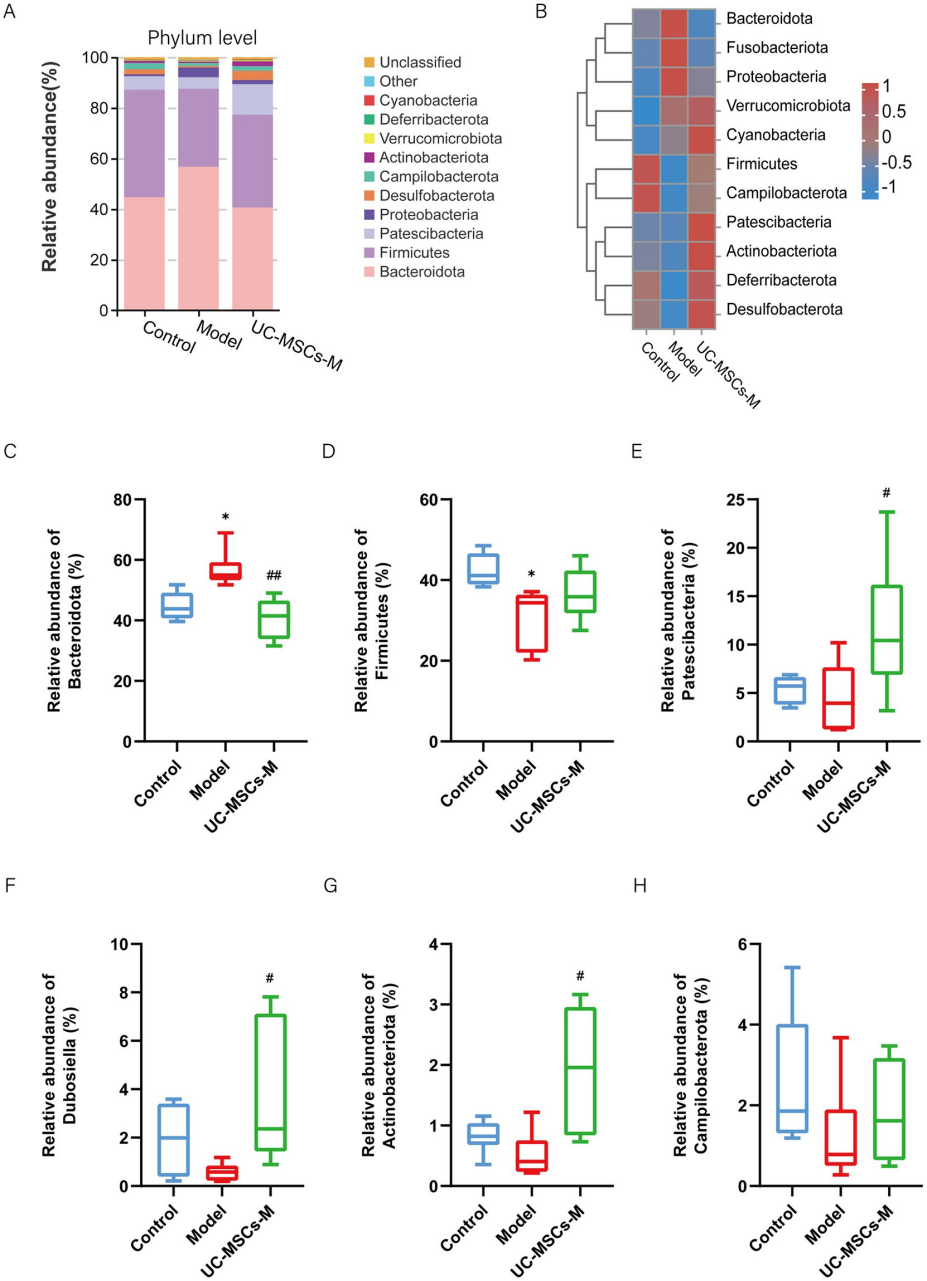
Mesenchymal stem cell therapy alters the gut microbiota (GM) composition at the phylum level in BLM-induced IPF model mice. (**A**) Bacterial taxa relative abundance of all the groups at the phylum level. (**B**) Heatmap of cluster stacking at the phylum level. The relative abundances of (**C**) *Bacteroidetes*. (**D**)*Firmicutes*. (**E**)*Pastesibateria*. (**F**)*Dubosiella*. (**G**)*Actinobacteriota*. (H) *Campilobacterota*. The data are expressed as the means±SDs (n = 6 for all the groups). (*p < 0.05, ** p < 0.01, ***p<0.001, ****p<0.0001 Model vs. Control; # p < 0.05, ## p < 0.01, ### p < 0.001, #### p < 0.0001 vs. Model).

At the phylum level, the evidently affected phylotypes of the GM were mainly involved with *Bacteroidota*, *Firmicutes*, *Pastescibacteria*, *Proteobacteria*, and *Campilobacterota* (Fig 3A and 3B). The proportion of *Bacteroidota* the richest phylotype in the Control group (Fig 3A), was most markedly increased from 44.74% in the Control group to 56.74% in the Model group (Fig 3C). However, treatment of BLM-induced IPF model mice with UC-MSCs in the UC-MSCs-M groups decreased the proportion of *Bacteroidota* in BLM-induced IPF model mice. In contrary, the modulation of *Firmicutes*, the second richest phylotype in the Control group (Fig 3D), had the opposite effect. BLM-induced IPF model mice in the Model group exhibited a slight decrease in the proportion of *Pastescibacteria*, whereas treatment of BLM-induced IPF model mice with UC-MSCs reduced the increase in the *Pastescibacteria* proportion in BLM-injured mice (Fig 3E). *Dubosiella* and *Actinobacteriota* showed similar patterns of change (Fig 3F and 3G). Moreover, the richness of *Campilobacterota* did not significantly differ among the groups (Fig 3H).

**Fig 4:**
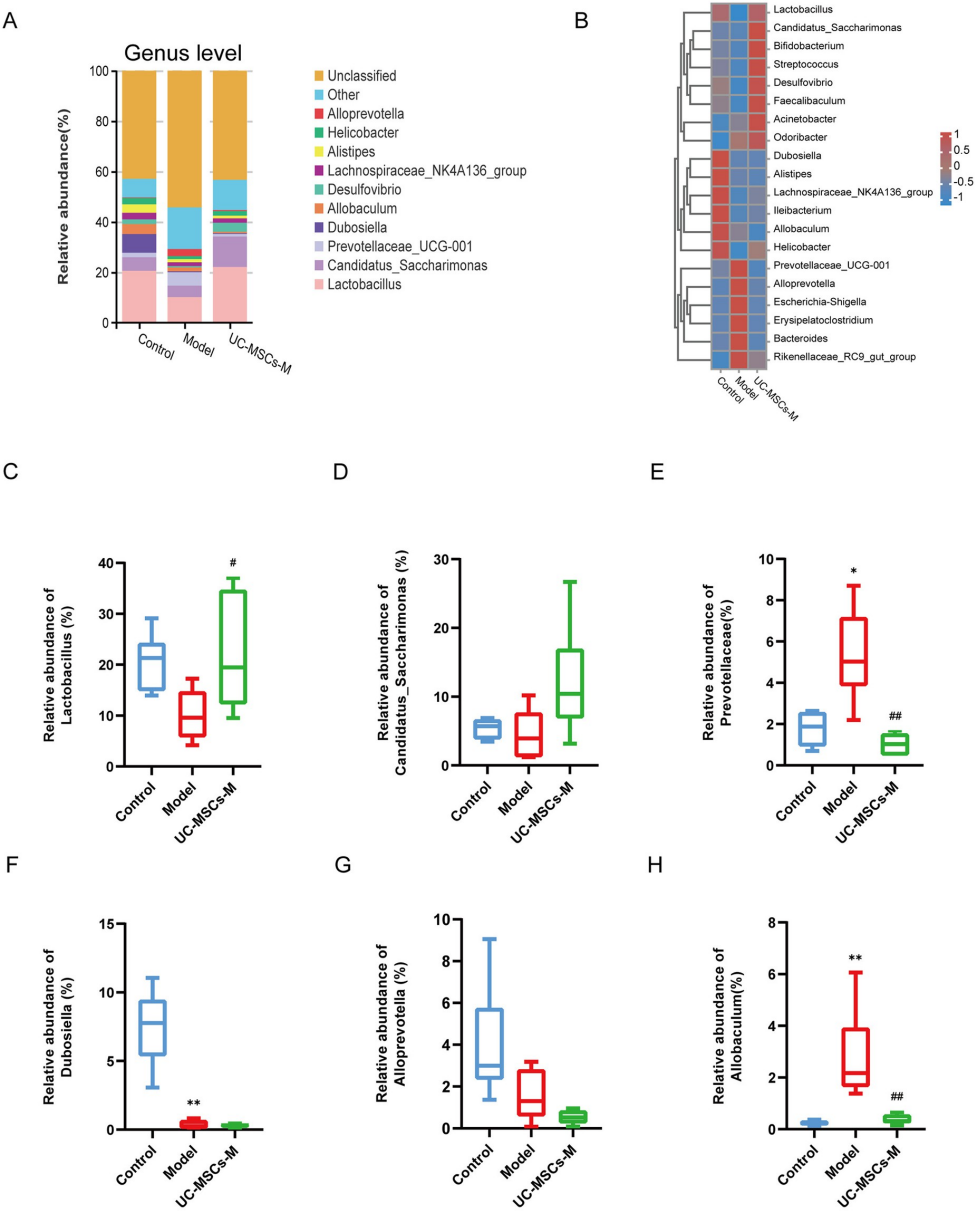
Mesenchymal stem cell therapy alters the gut microbiota (GM) composition at the genus level in BLM-induced IPF model mice. (**A**) Bacterial taxa relative abundance of all the groups at the genus level. (**B**) Heatmap of cluster stacking at the genus level. The relative abundances of (**C**) *Lactobacillus*. (**D**) *Candidatus_Saccharimonas*. (**E**) *Prevotellaceae*. (**F**) *Dubosiella*. (**G**) Desulfovibrio. (**H**) *Allobaculum*. The data are expressed as the means±SDs(n = 6 for all the groups). (* p < 0.05, ** p < 0.001 Model vs. Control; *** p < 0.001, ****p<0.0001, # p < 0.05, ## p < 0.01, ### p < 0.001, ####p<0.0001 vs. Model).

At the genus level, compared to the Control group, the Model group had lower abundances of *Lactobacillus*, *Allobaculum*, *Alistipes*, *Helicobacter*, *Dubosiella*, *Lachnospiraceae_NK4A136_group* and *Candidatus_Saccharimonas* (Fig 4A). The heatmap of cluster stacking revealed that UC-MSCs treatment switched the abundances of the following bacteria to levels similar to those in the Control group: *Desulfovibrio*, *Streptococcus*, *Bacteroides*, *Prevotellaceae_UCG-001*, *Alloprevotella*, *Escherichia-Shigella* (Fig 4B). The histogram also revealed that the relative abundances of these bacteria were in accordance with the above results (Fig 4C–4H). Thus, UC-MSCs treatment partly counteracted the influence of BLM on the abundance profile of the above genera.

Taken together, these results suggest that UC-MSCs regulate the bacterial composition in BLM-injured mice, which may be beneficial for ameliorating pulmonary fibrosis and lung damage.

### 2.6 Analysis of differences in microorganisms among the three groups

**Fig 5:**
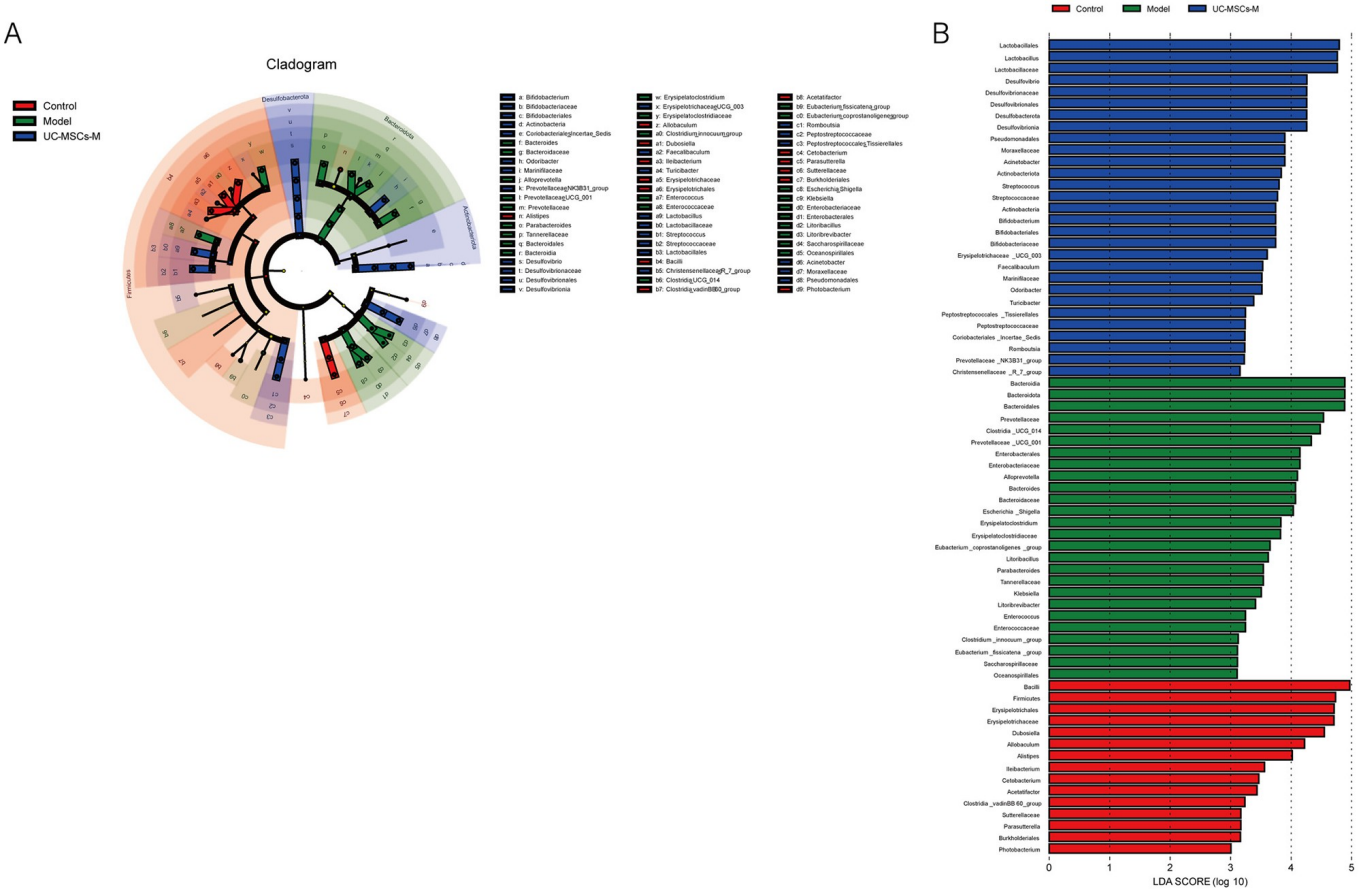
Analysis of the LEfSe. (**A**) Cladogram. The circles radiating from the inside out represent the classification level from boundary to genus. (**B**) Histogram of the LDA value distribution. The longer the length is, the greater the degree of influence.

To gain better insight into the impact of UC-MSCs intervention on the gut microbiota of mice, we conducted a linear discriminant analysis size effect (LEfSe). As shown in Fig 5A and 5B, the biomarkers with LDA values >3 and p < 0.05 were screened. The relative abundance of genera between groups was used to assess the impact of significantly different genera between groups. *Bacilli*, *Firmicutes*, *Erysipelotrichaceae*, *Erysipelotrichales*, and *Dubosiella* were enriched in the Control group. The genera *Bacteroidia*, *Prevotellaceae*, *Clostridia_UCG_014*, *Prevotellaceae_UCG_001*, and *Enterobacteriaceae* were enriched in the Model group. The UC-MSCs-M group exhibited increased relative abundances of *Lactobacillales*, *Desulfovibrio*, *Pseudomonadales*, *Moraxellaceae* and *Acinetobacter*.

### 2.7 Identification and screening of differentially abundant metabolites

**Fig 6:**
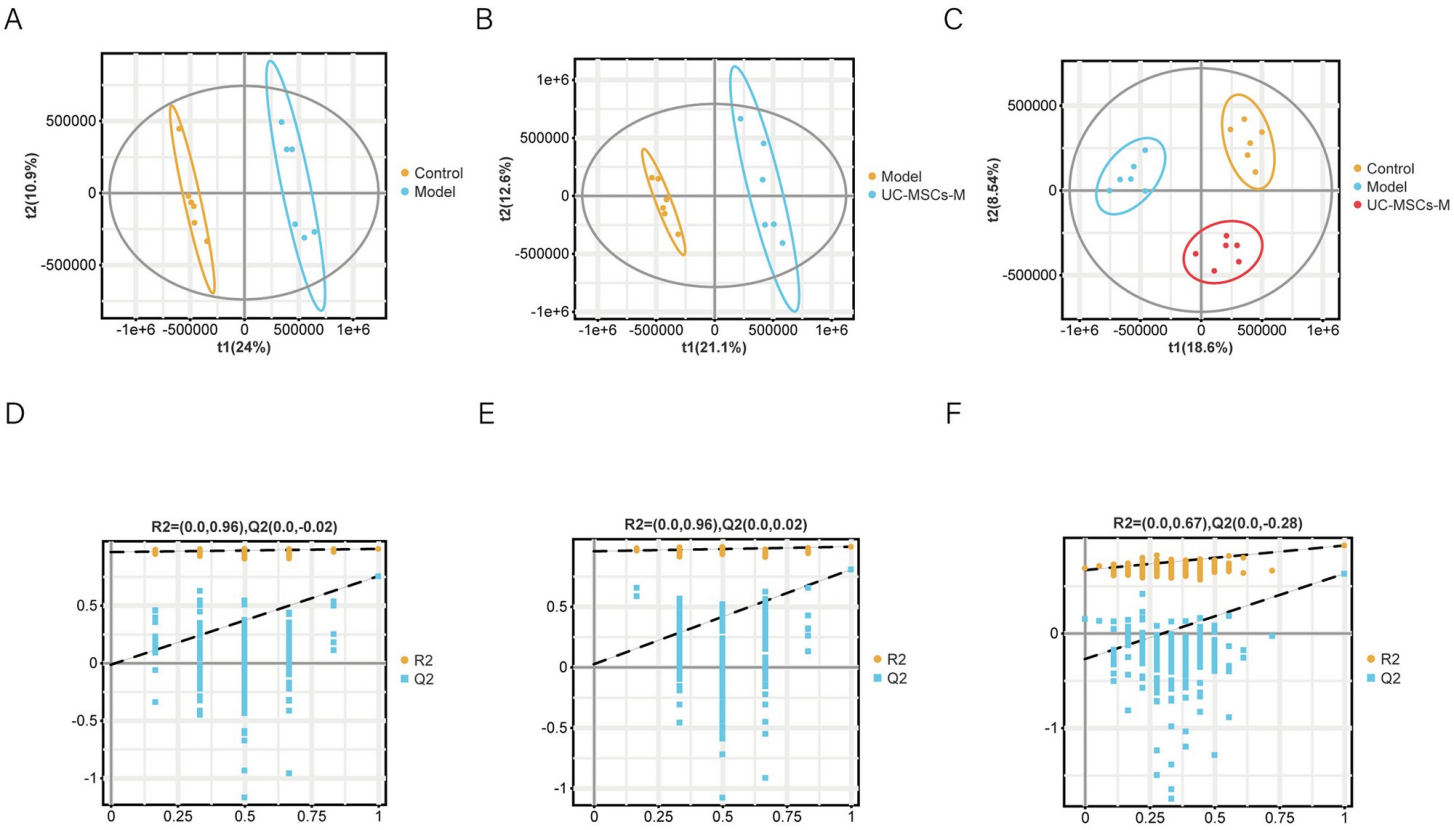
Score plots for the PLS-DA model and its corresponding permutation test plots. (**A**) PLS-DA diagrams of the Control and Model groups. (**B**) PLS-DA diagrams of the Model and UC-MSCs-M groups. (**C**) PLS-DA diagrams of the Control, Model and UC-MSCs-M groups. (**D-F**) Corresponding model validation plots.

On the basis of biochemical markers and histopathological findings, a medium dosage of UC-MSCs was chosen for metabolomics investigation. Metabolomic analysis of the mice fecal samples was conducted using UPLC-Q-TOF/MS, with a single quality control (QC) sample included for every 10 samples analyzed to monitor consistency during the injection process. Evaluation of the total ion chromatograms of the QC samples in both positive and negative ion modes revealed overlapping curves, supporting the reliability of the detection system. PLS-DA, a multivariate technique for supervised pattern recognition, was used in the analysis. The statistical analysis method utilized in this study identifies the most pertinent variables for grouping factors while mitigating the impact of extraneous factors. The PLS-DA scoring plots displayed in Fig 6A–6C clearly distinguish between the Model and Control groups and between the Model and UC-MSCs-M groups. Subsequent alignment testing revealed that the model’s R2 and Q2 values were lower than the initial values from left to right (Fig 6D–6F), indicating that the model exhibited good predictive capacity without overfitting.

**Fig 7:**
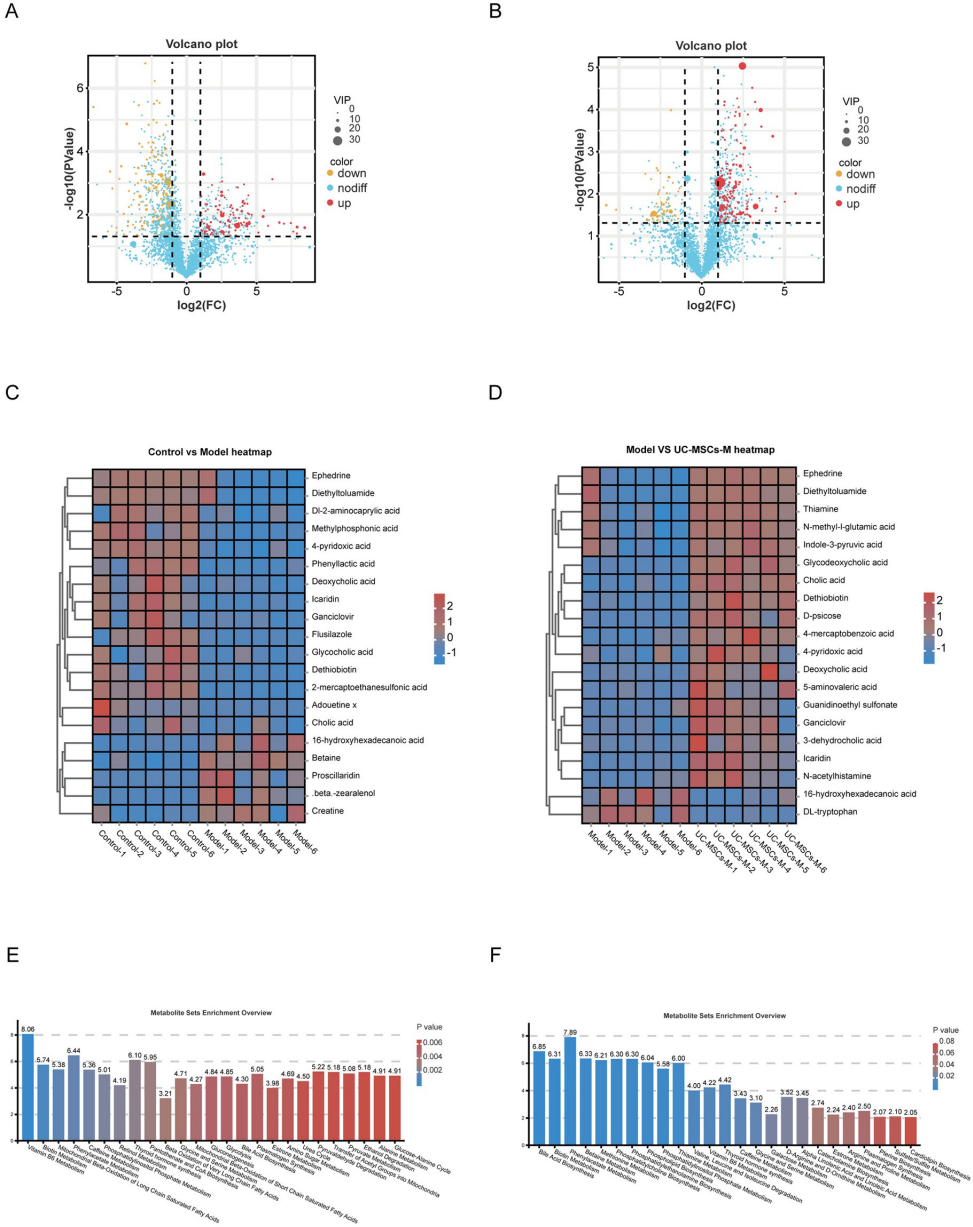
Differentially abundant metabolite identification and enrichment analysis based on the SMPDB database. Volcanic map of differentially abundant metabolites (**A**) Control vs. Model groups. (**B**) Model vs. UC-MSCs-M groups. The horizontal coordinate represents the fold change (log2FoldChange) of metabolites in different groups, and the vertical coordinate represents the significance level of the difference (-log10 Pvalue). Each point in the volcano plot represents a metabolite, where metabolites that are significantly upregulated are indicated by red dots and metabolites that are significantly downregulated are indicated by blue dots. (**C-D**) Heatmap of the clustering of differentially abundant metabolites. The colors from red to blue in the heatmap indicate the abundance of metabolites in each group of samples from high to low. Bar graph of significant enrichment of metabolic pathways based on SMPDB database analysis. (**E**) Control vs. Model groups. (**F**) Model vs. UC-MSCs-M groups. The horizontal coordinate is the metabolic pathway and the vertical coordinate is the degree of enrichment of differentially abundant metabolites in that pathway.

To further screen the differentially abundant metabolites, we analyzed them using PLS-DA and volcano plots (Fig 7A and 7B), and selected the metabolites that differed significantly between the Model group and the Control group, and between the Model group and the UC-MSCs-M group, and conditioned them on VIP > 1.5, P < 0.05, FC > 2 or FC < 0.5 (log_2_FC > 1 or log_2_FC < -1); subsequently, we used the HMDB database validate the differentially abundant metabolites. A total of 149 eligible differentially abundant metabolites were identified in the Model group compared with the Control group, of which 59 differentially abundant metabolites were upregulated and 90 differential metabolites were downregulate; whereas a total of 81 eligible differentially abundant metabolites were identified in the Model group, of which 49 differentially abundant metabolites were upregulated and 32 differentially abundant metabolites were downregulate compared with the UC-MSCs-M group. We selected the top 20 differentially abundant metabolites ranked by the VIP value for heatmap analysis, and the results revealed that 15 differentially abundant metabolites were significantly downregulated while 5 differentially abundant metabolites were upregulated in the Model group compared with those in the Control group, and 2 differentially abundant metabolites were significantly downregulated while 18 differentially abundant metabolites were upregulated compared with those in the UC-MSCs-M group. These metabolite species included Lipids and lipid-like molecules, Organoheterocyclic compounds, Organic acids and derivatives, Benzenoids (Fig 7C and 7D).

### 2.8 Enrichment analysis of differentially abundant metabolite pathways

To further explore the biological functions of the differentially abundant metabolites, we utilized the MetaboAnalystR package to perform MSEA (Metobolite Sets Enrichment Analysis) via The Small Molecule Pathway Database (SMPDB) database for all metabolites identified in the comparator group, which helped to identify and interpret important biological pathways involved in the pattern of changes in metabolite concentrations and to obtain information on pathways significantly enriched in the metabolites. Compared with those in the Control group the enriched pathways of the differentially abundant metabolites in the Model group were focused mainly on vitamin B6 metabolism, fatty acid oxidation, and alkaloid metabolism, whereas compared with that of the UC-MSCs-M group, the enriched pathways of the differentially abundant metabolites were focused mainly on bile acid metabolism, biotin metabolism, and biosynthesis of lipids as well as amino acids (Fig 7E and 7F).

### 2.9 Correlation analysis

**Fig 8:**
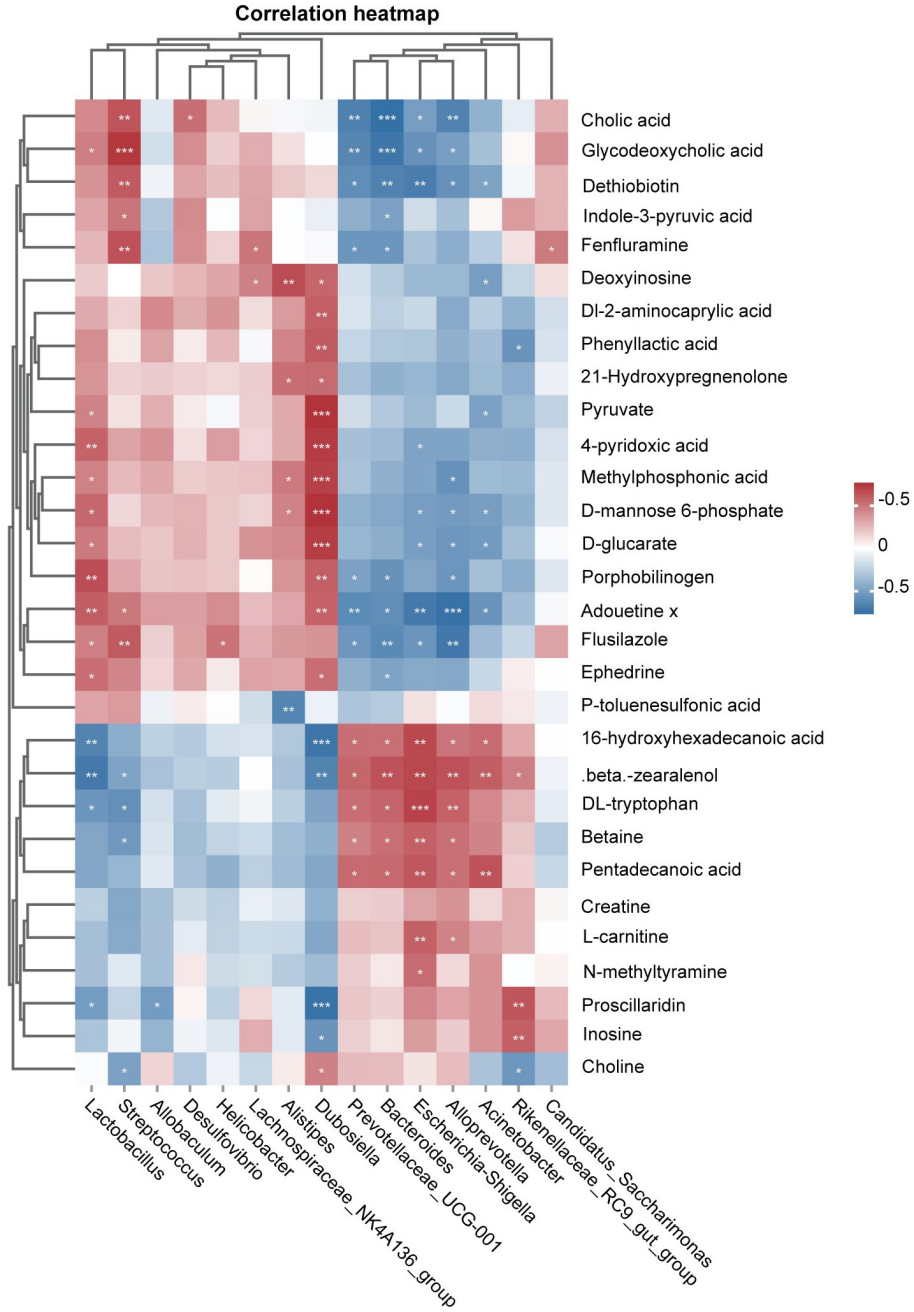
Spearman analysis of 30 significantly different metabolites and 15 key differentially abundant gut microbes. The gradient from red to blue indicates a positive correlation through negative correlation (*p<0.05, **p<0.01, ***p<0.001, ****p<0.0001).

Spearman correlation analysis was used to study the functional relationships among local biomarkers, differential bacteria, and biochemical parameters. As shown in Fig 8, *Prevotellaceae_UCG-001*, *Bacteroides*, *Escherichia-Shigella*, and *Alloprevotella* were all associated with 16-hydroxyhexadecanoic acid, beta. -zearalenol, DL- tryptophan, Betaine, and Pentadecanoic acid were positively correlated; and negatively correlated with Flusilazole, Adouetine x, Dethiobiotin, Glycodeoxycholic acid, and Cholic acid. *Lactobacillus*, *Dubosiella* was positively correlated with Pyruvate, 4-pyridoxic acid, Methylphosphonic acid, D-mannose 6-phosphate, D-glucarate, Porphobilinogen, Adouetine x, 16-hydroxyhexadecanoic acid and beta-zearalenol.

## 3. Discussion

Bleomycin induces pathological changes in the endobronchial buds, collagenous walls, and alveolar spaces that closely resemble the histological characteristics observed in patients with IPF. The use of bleomycin models offers several advantages, such as ease of manipulation, widespread availability, reproducibility, and alignment with key criteria for an effective animal model [20]. Therefore, bleomycin is often used to mimic the characteristics of human IPF lung injury. Stem cell therapies present a promising therapeutic approach for pulmonary fibrosis, and advancements in technology continue to expand the scope of potential treatments. MSCs are pivotal in the process of tissue regeneration and repair through their ability to differentiate into various cell types, exhibit immunogenic properties, and secrete anti-inflammatory factors to facilitate tissue healing [9, 21]. Furthermore, clinical studies have shown no adverse effects in the treatment of IPF via the use of MSCs derived from umbilical cords [22], which are easily isolated and expanded for further research on their therapeutic potential. The deposition of the extracellular matrix plays a crucial role in the pathogenesis of IPF, with collagen serving as a key component of the extracellular matrix. Hydroxyproline, a constituent of collagen, also plays a significant role in this process. Our study revealed a decrease in hydroxyproline content in mice treated with UC-MSCs compared with those with BLM-induced injury. Furthermore, UC-MSCs treatment led to improved survival rates and reduced lung fibrosis in the mice. These findings suggest that UC-MSCs may represent a promising therapeutic approach for treating IPF.

Persistent inflammation is a key characteristic of IPF, in which the compromised alveolar epithelium releases various cytokines and growth factors, such as TGF-β, leading to the transformation of fibroblasts into contractile myofibroblasts capable of generating ECM [23]. Conversely, the secretion of inflammatory mediators by activated fibroblasts/myofibroblasts facilitates fibrogenesis and attracts immune cells, thereby intensifying chronic inflammation [24]. The findings from ELISA analyses indicated increased levels of the BALF proinflammatory factors IL-1, TNF-α, and IL-6, as well as the profibrotic factor TGF-β1 in mice with BLM injury, which inconsistent with prior research [23, 25, 26]. The findings of our study indicate that UC-MSCs attenuate the inflammatory response in BLM-challenged mice. Interestingly, our results suggest that the efficacy of UC-MSCs does not exhibit a dose-dependent relationship, as evidenced by previous research demonstrating that low-dose MSC treatment yields nonsignificant effects while high-dose treatment may exacerbate the risk of pulmonary embolism and inflammation, as well as interfere with macrophage phagocytosis and diminish treatment efficacy [27–29]; however the exact mechanism remains unclear. Our research offers valuable insights into determining the optimal dosage of MSCs therapy; however, further investigations and explorations are necessary to fully understand the potential of MSCs in human therapeutic applications.

Previous studies have demonstrated that the gut microbiota plays a role in IPF and is associated with inflammation [30, 31]. Therefore, the gut microbiota has emerged as a new target for IPF treatment. Our study used 16S rDNA sequencing analysis to determine the effects of medium-dose UC-MSCs on the composition and structure of the gut microbiota in IPF mice. In this study, *BacteroidotaandFirmicutes* were the most abundant phylum of the gut microbiota in the Control group. The relative abundance of *Bacteroidota* was significantly increased in IPF model mice, whereas that of *Firmicutes* was significantly decreased. Treatment with UC-MSCs resulted in a significant decrease in the relative abundance of *Bacteroidota*, whereas the relative abundances of *Patescibacteria*, *Dubosiella*, and *Actinobacteriota* were significantly increased. At the genus level, the relative abundance of *Dubosiella* was significantly downregulated in the Model group, whereas the relative abundances of *Prevotellaceae_UCG-001* and *Allobaculum* was significantly upregylated. Treatment with UC-MSCs resulted in a significant downregulation of the relative abundances of *Prevotellaceae_UCG-001* and *Allobaculum*, and the relative abundances of *Lactobacillus* and *Desulfobacterota* were significantly upregulated in relative abundance. *Lactobacillus*, a probiotic classified within the phylum *Firmicutes* and commonly found in the gastrointestinal tract, serves an immunomodulatory function in the management of individuals with chronic respiratory conditions [32]. Previous research has demonstrated that *Lactobacillus* can translocate from the intestines to the lungs through the mesenteric lymphatic system or oropharyngeal reflux [33], stimulating lung macrophages and natural killer cells, and facilitating the recruitment of Treg cells to the lungs, thereby eliciting anti-inflammatory responses [34]. *Lactobacillus* can to secrete various short-chain fatty acids, including propionate, butyrate, and acetate [35], which are known for their potent anti-inflammatory properties that play a role in regulating the immune function of the host’s lungs [36–38]. Our findings revealed a decrease in the abundance of *Lactobacillus* in the Model group, which was subsequently increased following treatment with UC-MSCs. However, further investigations are neededto fully elucidate the underlying mechanism involved. *Desulfovibrio*, a sulfate-reducing bacterium, has been implicated in the pathogenesis of various diseases including Parkinson’s disease, inflammatory bowel disease, and bacteremia [39]. *Desulfovibrio* is known to carry out various biological functions, such as inflammation and oxidative stress, predominantly by generating the noxious gas hydrogen sulfide(H2S), and is generally regarded as a pathogenic bacterium [40]. However, our results showed that the abundance of *Desulfovibrio* instead decreased in the Model group of mice, which may be due to differences in climate, diet, and sampling techniques. A previous study revealed that *Desulfovibrio* is not always associated with adverse health effects [41], thus, the specific role *Desulfovibrio* plays in IPF needs to be further explored. *Prevotellaceae* is widespread in all parts of the body and plays an important role in maintaining human health; however, its association with disease is unclear [42]. Some studies have shown that *Prevotellaceae* can slow the progression of Parkinson’s disease through the production of short-chain fatty acids [43], whereas others have shown that *Prevotellaceae* can induce a TH2 immune response that exacerbates asthma [44]. The results revealed that the abundance of *Prevotellaceae* was significantly increased in our Model group of mice and significantly decresed after treatment, which, although the mechanism is not known, may provide some insights into the role it plays in different diseases. *Allobaculum*, is a gram-negative bacillus that induces the amplification of inflammatory intestinal T-helper 17 cells, compromises the intestinal barrier and contributes to the development of autoimmune hepatitis via the gut–liver axis [45]. Previous studies have also shown that *Allobaculum* may exacerbate leaky gut, leading to increased LPS production, which enters the brain via the gut–brain axis and exacerbates neuroinflammation in Alzheimer’s disease (AD) by exacerbating the production of Aβ in neurons and the activation of glial cells [46]. In our study, the abundance of *Allobaculum* increased in the Model group but decreased in the UC-MSCs group, which is consistent with the findings of previous studies showing that *Allobaculum* may promote the progression of inflammatory disease. However, in follow-up studies, it will be necessary to delve deeper into the precise function of *Allobaculum* in the context of lung disease. In summary, UC-MSCs can effectively inhibit inflammatory cell infiltration and pulmonary fibrosis by regulating the gut microbiota.

The findings of this study suggest a correlation between alterations in gut microbiota and metabolite levels in response to UC-MSC treatment in IPF model mice. Bile acids, known for their crucial role in intestinal nutrient absorption and lipid secretion, are also involved in maintaining homeostasis and exerting anti-inflammatory effects. Previous research has shown that bile acids can activate farnesoid X receptors (FXRs), thereby suppressing inflammation and fibrosis in various organs including the liver, kidneys, and intestines [47]. FXR activation has also been shown to inhibit the inflammatory response and to promote lung repair after lung injury [48]. The results of the present study revealed that the level of bile acids and their metabolites Deoxycholic acid, Glycodeoxycholic acid, and 3-dehydrocholic acid were down-regulated in the Model group, whereas they were increased after treatment, suggesting that bile acids and their metabolites may play a protective role in the treatment of IPF via the administration of UC-MSCs, which is in line with previous findings [49, 50]. In addition, according to the pathway enrichment results, the biotin synthesis pathway plays a key role in the treatment of IPF with UC-MSCs. Biotin is a water-soluble B-type vitamin that is involved in a variety of cellular metabolic pathways, such as gluconeogenesis, fatty acid synthesis, and fatty acid oxidation. Biotin deficiency may lead to increased expression of proinflammatory factors, immune dysfunction, and activation of related inflammatory pathways [51]. Previous studies have shown that biotin synthesis has an inhibitory effect on Mycobacterium abscessus in the lungs and affects the growth of Mycobacterium abscessus by influencing PH through supporting fatty acid remodeling and envelope mobility [52]; furthermore, the aggregation of inflammatory cells such as eosinophils, foam cells, and macrophages in the lungs of rats fed a biotin-deficient diet has been observed [53], suggesting that biotin has an impact on inflammatory responses. Our research offers insights into the biotin synthesis pathway as a potential treatment for IPF, yet further investigation is needed to fully elucidate the underlying mechanism involved. In recent years, the role of lipid metabolism in the progression of lung diseases has received much attention, such as the alteration of phospholipids and their metabolites in the plasma of patients with IPF, has received much attention [54]. Phosphatidylcholine and phosphatidylethanolamine, metabolites of glycerophospholipids, have previously been shown to promote pulmonary fibrosis by mediating microvascular injury and promoting fibroblast migration and proliferation [55, 56]. In our study, the biosynthesis of these two substances was also demonstrated to play a key role in the treatment of PF by UC-MSCs, which is consistent with previous findings [57]. Taken together, the survival of IPF mice treated with UC-MSCs may be attributed to changes in metabolites. However, the mechanism of metabolite changes remains to be investigated.

In recent years, it has become increasingly clear that the gut plays a crucial role in directing immune responses outside the local environment, including the lungs, and that small molecule compounds play a role in connecting the lung-gut axis [58]. In our research, we performed a Spearman correlation analysis of altered gut microbiota and potential metabolic markers. UC-MSCs have the ability to regulate the abandunce of *Prevotellaceae_UCG-001*, *Bacteroides*, *Escherichia-Shigella*, *Alloprevotella* and *Dubosiella*, as well as the ability to modulate 16-hydroxyhexadecanoic acid, Glycodeoxycholic acid, and Cholic acid, thereby regulating bile acid metabolic pathways and protecting lung tissues from inflammatory factors. However, further studies are needed to confirm these hypotheses. In conclusion, we generated a murine model of IPF using BLM and observed that UC-MSCs exhibit therapeutic efficacy in attenuating lung injury and decreasing the level of inflammatory mediators in vivo. Furthermore, we investigated alterations in the gut microbiota and fecal metabolites in conjunction with omics approaches to elucidate the underlying mechanisms and identify potential biomarkers for UC-MSCs therapy in IPF. Nevertheless, our study has certain limitations, including (1) The small sample size of animals utilized and the necessity for further validation through additional animal experiments and clinical studies. (2) The study lacked an evaluation of the efficacy of the Control+UC-MSCs group. (3) There is a lack of evidence that UC-MSCmediated protection against pulmonary fibrosis is attributable to alterations in the gut microbiome and its metabolites. Futhermore, the absence of sterile mice and fecal microbiota transplants hinders the confirmation of a causal relationship between the microbiota and pulmonary fibrosis, highlighting the need for a more sophisticated experimental design to elucidate the mechanism of UC-MSCs in treating IPF.

## 4. Conclusion

In summary, our study used a multifaceted approach including analysis of altered inflammatory responses, 16S rDNA sequencing, and metabolomics to explore the effects of the use of UC-MSCs on pulmonary fibrosis, providing a basis for further exploration of the pathology of IPF and the development of new treatment approaches. The results of this study suggest that UC-MSCs treatment attenuates the progression of PF via a mechanism that may prevent inflammation by restoring the diversity of the gut microbiota and its metabolite changes. However, we did not investigate in this how UC-MSCs modulate the interaction between the microenvironmental microbiota and metabolites, which will be further explored in the future.

## 5. Methods and materials

### 5.1 UC-MSCs preparation

The neonatal umbilical cords used for the isolation of hUC-MSCs were obtained from healthy donors according to standard protocols. Each donor signed the informed consent form, and the Second Affiliated Hospital ethics committee of Fujian Medical University approved the protocol for bone marrow harvesting (Approval No.2021395). The recruitment period ranged from October 1, 2021 and completed on December 1, 2022. in the cells at passage 3 (P3) were analyzed by flow cytometry with the following antibodies: CD11b/c (MA1-80091, Thermo Fisher Scientific), CD45 (ab10558, Abcam), HLA-DR (SAB4700731, Sigma-Aldrich), CD29 (10587-MM06, Sino Biological), CD44 (MA4400, Thermo Fisher Scientific), CD73 (10904-MM07, Sino Biological), CD90 (MA5-16671, Thermo Fisher Scientific), and CD105 (10149-R103, Sino Biological). Trilineage differentiation of the isolated cells was assessed for their adipogenic, chondrogenic, and osteogenic differentiation potential using differentiation kits (Cyagen) according to the instructions. Here, the UC-MSCs used in this process were CD29^+^, CD44^+^, CD73^+^, CD90^+^, CD105^+^, CD11b/c^-^, CD45^-^, HLA-DR^-^ and with the potential of lipogenic, chondrogenic, as well as osteogenic (S1 Fig).

### 5.2 Animal

Female 8–10-week -old C57BL/6 mice were purchased from Shanghai SLAC Laboratory Animal Co. Ltd. (Shanghai China). All the mice were housed in an approved animal facility and cared for by a licensed veterinarian and supervised staff under a 12-hour light/12-hour dark cycle with access to food and water ad libitum. All experimental protocols were approved by the Institutional Animal Care and Use Committee of the Second Affiliated Hospital of Fujian Medical University (Approval No.2021395). The animal experiments were conducted from April 2023 to June 2023.

### 5.3 Animal experimental treatment and UC-MSCs administration

Mice (n = 75) were randomized into five groups. Group 1 was normal control group (Control), the mice in the control group were treated with 100 μL of sterile saline. Group 2 was model group (Model), in which the mice were anesthetized with pentobarbital sodium and intubated with a 21G atraumatic cannula for intratracheal instillation of 2U/Kg. Groups 3–5 included UC-MSC-low, UC-MSC-medium, and UC-MSC-high treatment groups. BLM-induced IPF mice were intravenously administered UC-MSCs on Day 7, and the numbers of UC-MSCs injected through the vein were 2.5×10^5^ cells, 5×10^5^ cells, and 1×10^6^ cells in Groups 3, 4, and 5, respectively. The mice were maintained until they were euthanized on Day 21. Then, cecal content samples were randomly collected from each group (n = 6 for all groups) as soon as possible and immediately stored in liquid nitrogen for further analyses. The body weights of the mice were measured every other day. At the endpoint (Day 21), the mice were sacrificed with 3% sodium pentobarbital, and all efforts were made to minimize discomfort and pain.

### 5.4 Bioluminescence imaging of Fluc-labeled UC-MSCs

For bioluminescence imaging (BLI), firefly luciferase (Fluc) was used for MSCs. For intravital imaging, IPF model mice were established via intratracheal injection of BLM. Next, IPF models received intravenous injections of Fluc-labeled UC-MSCs in a volume of 100 μL. IPF model mice administered Fluc-labeled UC-MSCs were imaged using the AniView Kirin Imaging System (Guangzhou Biolight Biotechnology Co., Ltd.) after intraperitoneal injection of the D-luciferin substrate (150 mg/kg; Biosynth International, USA). The results of BLI revealed that the fluorescence signal was strongest in the lungs and peaked on Day 1. Thereafter, the fluorescence signal became weaker and disappeared on Day 5, and these results indicated that UC-MSCs migrated mainly to the lungs (S2 Fig).

### 5.5 Lung morphology analysis and hydroxyproline assay

The mice were sacrificed on the 21st day after the stimulation with BLM or saline and the right lung tissues of the mice were dissected. First, the mice were lavaged three times with 500 μL of phosphate-buffered saline (PBS) and fixed by injecting 500 μL with 4% paraformaldehyde. After fixation by 4% paraformaldehyde for 24 h and gradient dehydration with alcohol, the lung tissues were embedded in paraffin. They were cut into 5 μm sections from the frontal, middle, and posterior coronal planes. HE and MT staining were performed according to previously described methods for histology analysis. To determine fibrosis severity, tissue sections were scanned using a digital pathological image scanner. Histologic quantification was conducted using the Ashcroft score based on the H&E and Masson staining in a blinded manner [59].

Hydroxyproline is a significant component of collagen in the lung tissues. To determine the hydroxyproline content, the mice were dissected, the surface blood were drained. We then weighed 50 mg of wet right lung tissue and determined the hydroxyproline content according to the content assay kit instructions (Jiancheng Bioengineering Institute, Nanjing, China).

### 5.6 Bronchoalveolar lavage fluid (BALF) collection and measurement of cytokine levels via enzyme-linked immunosorbent assay (ELISA)

After euthanasia, the thorax of each mouse was opened to expose the trachea, and the right trachea and lung lobes were ligated. The left lung was lavaged three times with 0.9 ml of PBS. The BALF was centrifuged at 12,000×g for 5 min, after which the supernatant was collected and stored at -80°C. The protein concentration was detected via the BCA method. Fifty micrograms of protein were measured to evaluate the levels of TNF-α, IL-1β, IL-6, and TGF-β1 via ELISA kits (BOSTER, Wuhan, China) following the manufacturer’s instructions.

### 5.7 Analysis of the intestinal microbiota

Microbial DNA was extracted using the HiPure Soil DNA Extraction Kit (Magen, Guangzhou, China) following the manufactuer’s instructions. The 16S rDNA V3–V4 region was amplified by PCR (95°C for 5 min, then 95°C for 1 min, 60°C for 1 min, 72°C for 1 min for 30 cycles, and finally 72°C for 7 min) using the following primers: 341F: CCTACGGGNGGCWGCAG; 806R: GGACTACHVGGGTATCTAAT. Amplicons were extracted from 2% agarose gels, purified using the AxyPrep DNA Gel Extraction Kit (Axygen Biosciences, Union City, CA, USA) according to the manufacturer’s instructions, and quantified using the ABI StepOnePlus Real-Time PCR System (Life Technologies, Foster City, CA, USA). The purified amplicons were subjected to double-end sequencing on the Illumina platform according to standard operations (PE250). The raw reads were further filtered using FASTP (version 0.18.0). The raw data from the Illumina platform were filtered using FASTP (version 0.18.0). Clean reads were merged into tags using FLASH (version 1.2.11) at a minimum overlap of 10 bp and a maximum mismatch rate threshold of 2%. The raw tags were than quality filtered, and chimeric sequences were removed to acquire effective tags, which were clustered into operational taxonomic units (OTUs) with ≥97% identity cutoff using UPARSE software (version 9.2.64). PCA was performed in the R project Vegan package (version 2.5.3). The Chao1 and ACE richness indices were determined with QIIME software (version 1.9.1, University of Colorado, Denver, CO, USA). The dominant bacteria were analyzed mainly at the phylum and genus levels via the R project. Venn analysis was performed via the language VennDiagram package (version 1.6.16) and the R language UpSetR package (version 1.3.3) to analyze shared endemic species/OTUs/ASVs between groups. Species comparisons among in different groups was conducted via Welch’s t-test in the R project Vegan package (version 2.5.3). The heatmap of cluster stacking was calculated using the R package and generated via OmicsMart (Gene Denovo Biotechnology Co. Ltd., Guangzhou, China), a dynamic real-time interactive platform for data analysis.

### 5.8 Untargeted metabolome LC-MS/MS experimental methods and data analysis

#### 5.8.1 UHPLC-Q-TOF

The samples were selected as described above. The samples were separated on an Agilent 1290 Infinity LC ultrahigh-performance liquid chromatography (UHPLC) HILIC column; the column temperature was 25°C; the flow rate was 0.5 mL/min; the injection volume was 2 μL; the mobile phases consisted of A: water + 25 mM ammonium acetate + 25 mM ammonia, and B: acetonitrile; and the gradient elution program was as follows: 0–0.5 min. 95% B; 0.5–7 min, B varied linearly from 95% to 65%; 7–8 min, B varied linearly from 65% to 40%; 8–9 min, B was maintained at 40%; 9–9.1 min, B varied linearly from 40% to 95%; 9.1–12 min, B was maintained at 95%; the samples were placed at 4°C for the whole analysis process. The samples were kept in a 4°C autosampler throughout the analysis. To avoid the influence of fluctuations in the instrumental detection signal, the samples were analyzed continuously in a random order. QC samples were inserted into the sample queue for monitoring and evaluating the stability of the system and the reliability of the experimental data.

An AB Triple TOF 6600 mass spectrometer was used for the primary and secondary spectra of the samples. The ESI source conditions after HILIC chromatographic separation were as follows: Ion Source Gas1 (Gas1): 60, Ion Source Gas2 (Gas2): 60, Curtain gas (CUR): 30, source temperature: 600°C, IonSapary Voltage product ion scan accumulation time 0.05 s/spectra. The secondary mass spectra were obtained by information dependent acquisition (IDA) in high sensitivity mode, with a declustering potential (DP) 05 s/spectra. The secondary mass spectra were obtained by information dependent acquisition (IDA) and in high- sensitivity mode. The declustering potential (DP)was ±60 V (positive and negative modes), Collision Energy: 35±15eV, IDA settings were as follows Exclude isotopes within 4 Da, Candidate ions to monitor per cycle: 10.

#### 5.8.2 Metabolomics data analysis

The raw MS data were converted to MzXML files using ProteoWizard MSConvert before be imported into freely available XCMS software. The data obtained from XCMS extraction were first checked for completeness, metabolites with more than 50% missing values within the group were removed from subsequent analysis, null values were KNN filled, extreme values were removed, and finally, the data were normalized for total peak area to ensure that they could be compared for parallelism between samples and between metabolites. Orthogonal partial least squares identification analysis (OPLS-DA) was used to predict the reliability and stability of the model for rat stool sample data, and partial least squares identification analysis (PLS-DA) was used to assess the differences between groups. We combined multivariate statistical analysis of PLS-DA VIP values and univariate statistical analysis of T-test P-values to screen for significantly different metabolites between the different comparison groups. The thresholds for significant differences were a VIP ≥ 1.5 in the PLS-DA model and a ttest P<0.05. After the metabolites were identified, metabolite pathway enrichment analysis was performed on the differentially abundant metabolites via SMPDB, and pathways with Q value ≤ 0.05 were defined as pathways significantly enriched in the differentially expressed metabolites after correction for multiple testing. Significant enrichment of pathways allow for identification of the most important biochemical metabolic pathways and signaling pathways associated with the differentially expressed metabolites.

### 5.9 Statistical analysis

The data are presented as the means ± SDs. The significance of the differences was determined by analysis of variance (ANOVA). Histograms were created using Graph Pad Prism 9 software (San Diego, CA, USA). Bioinformatics analysis, including species taxonomy, richness and diversity analyses, was performed via OmicsMart. A p-value < 0.05 indicated a statistically significant difference.

## Supporting information

S1 Fig Identification of UC-MSCs. (A) Cells were analyzed by flow cytometry with the following antibodies: anti-CD11b/c, anti-CD45, anti-HLA-DR, anti-CD29, anti-CD44, anti-CD73, anti-CD90, and anti-CD105. (B) Identification of the potential of UC-MSCs for lipogenic, chondrogenic and osteogenic differentiation.
(TIF)

S2 Fig Evaluation of UC-MSC delivery through intravenous administration. Fluorescence imaging of different *ex vivo* organs (brain, lung, heart, liver, spleen and kidney) 24 h after the administration of UC-MSCs.
(TIF)

S1 Data (ZIP)

## References

[1] Meyer KC. Pulmonary fibrosis, part I: epidemiology, pathogenesis, and diagnosis. Expert Rev Respir Med. 2017;11(5):343–59. Epub 2017/03/28. doi: 10.1080/17476348.2017.1312346 . 28345383

[2] King TE Jr., Pardo A, Selman M. Idiopathic pulmonary fibrosis. Lancet. 2011;378(9807):1949–61. Epub 2011/07/02. doi: 10.1016/S0140-6736(11)60052-4 . 21719092

[3] Bonella F, Spagnolo P, Ryerson C. Current and Future Treatment Landscape for Idiopathic Pulmonary Fibrosis. Drugs. 2023;83(17):1581–93. Epub 2023/10/26. doi: 10.1007/s40265-023-01950-0 37882943 PMC10693523

[4] Noble PW, Barkauskas CE, Jiang D. Pulmonary fibrosis: patterns and perpetrators. J Clin Invest. 2012;122(8):2756–62. Epub 2012/08/02. doi: 10.1172/JCI60323 . 22850886 PMC3408732

[5] Ding DC, Shyu WC, Lin SZ. Mesenchymal stem cells. Cell Transplant. 2011;20(1):5–14. Epub 2011/03/15. doi: 10.3727/096368910X . 21396235

[6] Can A, Karahuseyinoglu S. Concise review: human umbilical cord stroma with regard to the source of fetus-derived stem cells. Stem Cells. 2007;25(11):2886–95. Epub 2007/08/11. doi: 10.1634/stemcells.2007-0417 . 17690177

[7] Heukels P, Moor CC, von der Thüsen JH, Wijsenbeek MS, Kool M. Inflammation and immunity in IPF pathogenesis and treatment. Respir Med. 2019;147:79–91. Epub 2019/02/02. doi: 10.1016/j.rmed.2018.12.015 . 30704705

[8] Racanelli AC, Kikkers SA, Choi AMK, Cloonan SM. Autophagy and inflammation in chronic respiratory disease. Autophagy. 2018;14(2):221–32. Epub 2017/11/14. doi: 10.1080/15548627.2017.1389823 . 29130366 PMC5902194

[9] Choi SM, Mo Y, Bang JY, Ko YG, Ahn YH, Kim HY, et al. Classical monocyte-derived macrophages as therapeutic targets of umbilical cord mesenchymal stem cells: comparison of intratracheal and intravenous administration in a mouse model of pulmonary fibrosis. Respir Res. 2023;24(1):68. Epub 2023/03/05. doi: 10.1186/s12931-023-02357-x . 36870972 PMC9985859

[10] Choi M, Ban T, Rhim T. Therapeutic use of stem cell transplantation for cell replacement or cytoprotective effect of microvesicle released from mesenchymal stem cell. Mol Cells. 2014;37(2):133–9. Epub 2014/03/07. doi: 10.14348/molcells.2014.2317 . 24598998 PMC3935626

[11] Ma PJ, Wang MM, Wang Y. Gut microbiota: A new insight into lung diseases. Biomed Pharmacother. 2022;155:113810. Epub 2022/10/23. doi: 10.1016/j.biopha.2022.113810 . 36271581

[12] Mercader-Barceló J, Truyols-Vives J, Río C, López-Safont N, Sala-Llinàs E, Chaplin A. Insights into the Role of Bioactive Food Ingredients and the Microbiome in Idiopathic Pulmonary Fibrosis. Int J Mol Sci. 2020;21(17). Epub 2020/08/28. doi: 10.3390/ijms21176051 . 32842664 PMC7503951

[13] Wei Y, Qi M, Liu C, Li L. Astragalus polysaccharide attenuates bleomycin-induced pulmonary fibrosis by inhibiting TLR4/ NF-κB signaling pathway and regulating gut microbiota. Eur J Pharmacol. 2023;944:175594. Epub 2023/02/23. doi: 10.1016/j.ejphar.2023.175594 . 36804541

[14] Gurczynski SJ, Lipinski JH, Strauss J, Alam S, Huffnagle GB, Ranjan P, et al. Horizontal transmission of gut microbiota attenuates mortality in lung fibrosis. JCI Insight. 2023;9(1). Epub 2023/11/28. doi: 10.1172/jci.insight.164572 . 38015634 PMC10911107

[15] Budden KF, Gellatly SL, Wood DL, Cooper MA, Morrison M, Hugenholtz P, et al. Emerging pathogenic links between microbiota and the gut-lung axis. Nat Rev Microbiol. 2017;15(1):55–63. Epub 2016/11/01. doi: 10.1038/nrmicro.2016.142 . 27694885

[16] Madan JC, Koestler DC, Stanton BA, Davidson L, Moulton LA, Housman ML, et al. Serial analysis of the gut and respiratory microbiome in cystic fibrosis in infancy: interaction between intestinal and respiratory tracts and impact of nutritional exposures. mBio. 2012;3(4). Epub 2012/08/23. doi: 10.1128/mBio.00251-12 . 22911969 PMC3428694

[17] Zhang D, Li S, Wang N, Tan HY, Zhang Z, Feng Y. The Cross-Talk Between Gut Microbiota and Lungs in Common Lung Diseases. Front Microbiol. 2020;11:301. Epub 2020/03/12. doi: 10.3389/fmicb.2020.00301 . 32158441 PMC7052046

[18] Su G, Wang H, Bai J, Chen G, Pei Y. A Metabonomics Approach to Drug Toxicology in Liver Disease and its Application in Traditional Chinese Medicine. Curr Drug Metab. 2019;20(4):292–300. Epub 2019/01/02. doi: 10.2174/1389200220666181231124439 . 30599107

[19] Hong W, Mo Q, Wang L, Peng F, Zhou Y, Zou W, et al. Changes in the gut microbiome and metabolome in a rat model of pulmonary arterial hypertension. Bioengineered. 2021;12(1):5173–83. Epub 2021/08/19. doi: 10.1080/21655979.2021.1952365 . 34405758 PMC8806624

[20] Ishida Y, Kuninaka Y, Mukaida N, Kondo T. Immune Mechanisms of Pulmonary Fibrosis with Bleomycin. Int J Mol Sci. 2023;24(4). Epub 2023/02/26. doi: 10.3390/ijms24043149 . 36834561 PMC9958859

[21] Mahmoudi T, Abdolmohammadi K, Bashiri H, Mohammadi M, Rezaie MJ, Fathi F, et al. Hydrogen Peroxide Preconditioning Promotes Protective Effects of Umbilical Cord Vein Mesenchymal Stem Cells in Experimental Pulmonary Fibrosis. Adv Pharm Bull. 2020;10(1):72–80. Epub 2020/02/01. doi: 10.15171/apb.2020.009 . 32002364 PMC6983995

[22] Zhang C, Yin X, Zhang J, Ao Q, Gu Y, Liu Y. Clinical observation of umbilical cord mesenchymal stem cell treatment of severe idiopathic pulmonary fibrosis: A case report. Exp Ther Med. 2017;13(5):1922–6. Epub 2017/06/02. doi: 10.3892/etm.2017.4222 . 28565787 PMC5443299

[23] Kishore A, Petrek M. Roles of Macrophage Polarization and Macrophage-Derived miRNAs in Pulmonary Fibrosis. Front Immunol. 2021;12:678457. Epub 2021/09/08. doi: 10.3389/fimmu.2021.678457 . 34489932 PMC8417529

[24] Kendall RT, Feghali-Bostwick CA. Fibroblasts in fibrosis: novel roles and mediators. Front Pharmacol. 2014;5:123. Epub 2014/06/07. doi: 10.3389/fphar.2014.00123 . 24904424 PMC4034148

[25] Zhao X, Wu J, Yuan R, Li Y, Yang Q, Wu B, et al. Adipose-derived mesenchymal stem cell therapy for reverse bleomycin-induced experimental pulmonary fibrosis. Sci Rep. 2023;13(1):13183. Epub 2023/08/15. doi: 10.1038/s41598-023-40531-9 . 37580529 PMC10425426

[26] Periera-Simon S, Xia X, Catanuto P, Coronado R, Kurtzberg J, Bellio M, et al. Anti-fibrotic effects of different sources of MSC in bleomycin-induced lung fibrosis in C57BL6 male mice. Respirology. 2021;26(2):161–70. Epub 2020/08/28. doi: 10.1111/resp.13928 . 32851725

[27] Liu YY, Chiang CH, Hung SC, Chian CF, Tsai CL, Chen WC, et al. Hypoxia-preconditioned mesenchymal stem cells ameliorate ischemia/reperfusion-induced lung injury. PLoS One. 2017;12(11):e0187637. Epub 2017/11/09. doi: 10.1371/journal.pone.0187637 . 29117205 PMC5678873

[28] Xia C, Chang P, Zhang Y, Shi W, Liu B, Ding L, et al. Therapeutic effects of bone marrow-derived mesenchymal stem cells on radiation-induced lung injury. Oncol Rep. 2016;35(2):731–8. Epub 2016/01/01. doi: 10.3892/or.2015.4433 . 26717975

[29] Preda MB, Neculachi CA, Fenyo IM, Vacaru AM, Publik MA, Simionescu M, et al. Short lifespan of syngeneic transplanted MSC is a consequence of in vivo apoptosis and immune cell recruitment in mice. Cell Death Dis. 2021;12(6):566. Epub 2021/06/03. doi: 10.1038/s41419-021-03839-w . 34075029 PMC8169682

[30] Liu JQ, Zhou HB, Bai WF, Wang J, Li Q, Fan LY, et al. Assessment of progression of pulmonary fibrosis based on metabonomics and analysis of intestinal microbiota. Artif Cells Nanomed Biotechnol. 2024;52(1):201–17. Epub 2024/03/15. doi: 10.1080/21691401.2024.2326616 . 38488151

[31] Yang J, Shi X, Gao R, Fan L, Chen R, Cao Y, et al. Polydatin alleviates bleomycin-induced pulmonary fibrosis and alters the gut microbiota in a mouse model. J Cell Mol Med. 2023;27(23):3717–28. Epub 2023/09/04. doi: 10.1111/jcmm.17937 . 37665061 PMC10718135

[32] Rastogi S, Singh A. Gut microbiome and human health: Exploring how the probiotic genus Lactobacillus modulate immune responses. Front Pharmacol. 2022;13:1042189. Epub 2022/11/11. doi: 10.3389/fphar.2022.1042189 . 36353491 PMC9638459

[33] Du T, Lei A, Zhang N, Zhu C. The Beneficial Role of Probiotic Lactobacillus in Respiratory Diseases. Front Immunol. 2022;13:908010. Epub 2022/06/18. doi: 10.3389/fimmu.2022.908010 . 35711436 PMC9194447

[34] Zhang J, Ma JY, Li QH, Su H, Sun X. Lactobacillus rhamnosus GG induced protective effect on allergic airway inflammation is associated with gut microbiota. Cell Immunol. 2018;332:77–84. Epub 2018/08/12. doi: 10.1016/j.cellimm.2018.08.002 . 30097177

[35] Espírito Santo C, Caseiro C, Martins MJ, Monteiro R, Brandão I. Gut Microbiota, in the Halfway between Nutrition and Lung Function. Nutrients. 2021;13(5). Epub 2021/06/03. doi: 10.3390/nu13051716 . 34069415 PMC8159117

[36] Berni Canani R, De Filippis F, Nocerino R, Laiola M, Paparo L, Calignano A, et al. Specific Signatures of the Gut Microbiota and Increased Levels of Butyrate in Children Treated with Fermented Cow’s Milk Containing Heat-Killed Lactobacillus paracasei CBA L74. Appl Environ Microbiol. 2017;83(19). Epub 2017/07/25. doi: 10.1128/AEM.01206-17 . 28733284 PMC5601345

[37] Li L, Fang Z, Liu X, Hu W, Lu W, Lee YK, et al. Lactobacillus reuteri attenuated allergic inflammation induced by HDM in the mouse and modulated gut microbes. PLoS One. 2020;15(4):e0231865. Epub 2020/04/22. doi: 10.1371/journal.pone.0231865 . 32315360 PMC7173794

[38] Chen L, Zhou X, Wang Y, Wang D, Ke Y, Zeng X. Propionate and Butyrate Produced by Gut Microbiota after Probiotic Supplementation Attenuate Lung Metastasis of Melanoma Cells in Mice. Mol Nutr Food Res. 2021;65(15):e2100096. Epub 2021/06/02. doi: 10.1002/mnfr.202100096 . 34061433

[39] Singh SB, Carroll-Portillo A, Lin HC. Desulfovibrio in the Gut: The Enemy within? Microorganisms. 2023;11(7). Epub 2023/07/29. doi: 10.3390/microorganisms11071772 . 37512944 PMC10383351

[40] Singh SB, Lin HC. Hydrogen Sulfide in Physiology and Diseases of the Digestive Tract. Microorganisms. 2015;3(4):866–89. Epub 2015/01/01. doi: 10.3390/microorganisms3040866 . 27682122 PMC5023273

[41] Chen YR, Jing QL, Chen FL, Zheng H, Chen LD, Yang ZC. Desulfovibrio is not always associated with adverse health effects in the Guangdong Gut Microbiome Project. PeerJ. 2021;9:e12033. Epub 2021/09/02. doi: 10.7717/peerj.12033 . 34466295 PMC8380029

[42] Tett A, Pasolli E, Masetti G, Ercolini D, Segata N. Prevotella diversity, niches and interactions with the human host. Nat Rev Microbiol. 2021;19(9):585–99. Epub 2021/05/30. doi: 10.1038/s41579-021-00559-y . 34050328 PMC11290707

[43] Heravi FS, Naseri K, Hu H. Gut Microbiota Composition in Patients with Neurodegenerative Disorders (Parkinson’s and Alzheimer’s) and Healthy Controls: A Systematic Review. Nutrients. 2023;15(20). Epub 2023/10/28. doi: 10.3390/nu15204365 . 37892440 PMC10609969

[44] Wang Z, Lai Z, Zhang X, Huang P, Xie J, Jiang Q, et al. Altered gut microbiome compositions are associated with the severity of asthma. J Thorac Dis. 2021;13(7):4322–38. Epub 2021/08/24. doi: 10.21037/jtd-20-2189 . 34422359 PMC8339736

[45] Wang K, Wu W, Jiang X, Xia J, Lv L, Li S, et al. Multi-Omics Analysis Reveals the Protection of Gasdermin D in Concanavalin A-Induced Autoimmune Hepatitis. Microbiol Spectr. 2022;10(5):e0171722. Epub 2022/08/17. doi: 10.1128/spectrum.01717-22 . 35972273 PMC9602755

[46] Lu S, Xu S, Chen L, Deng Y, Feng J. Periplaneta americana Extract Pretreatment Alleviates Oxidative Stress and Inflammation and Increases the Abundance of Gut Akkermansia muciniphila in Diquat-Induced Mice. Antioxidants (Basel). 2022;11(9). Epub 2022/09/24. doi: 10.3390/antiox11091806 . 36139880 PMC9495987

[47] Chiang JY. Bile acid metabolism and signaling. Compr Physiol. 2013;3(3):1191–212. Epub 2013/07/31. doi: 10.1002/cphy.c120023 . 23897684 PMC4422175

[48] Vignozzi L, Morelli A, Cellai I, Filippi S, Comeglio P, Sarchielli E, et al. Cardiopulmonary protective effects of the selective FXR agonist obeticholic acid in the rat model of monocrotaline-induced pulmonary hypertension. J Steroid Biochem Mol Biol. 2017;165(Pt B):277–92. Epub 2016/07/19. doi: 10.1016/j.jsbmb.2016.07.004 . 27425465

[49] Comeglio P, Morelli A, Adorini L, Maggi M, Vignozzi L. Beneficial effects of bile acid receptor agonists in pulmonary disease models. Expert Opin Investig Drugs. 2017;26(11):1215–28. Epub 2017/09/28. doi: 10.1080/13543784.2017.1385760 . 28949776

[50] Comeglio P, Filippi S, Sarchielli E, Morelli A, Cellai I, Corcetto F, et al. Anti-fibrotic effects of chronic treatment with the selective FXR agonist obeticholic acid in the bleomycin-induced rat model of pulmonary fibrosis. J Steroid Biochem Mol Biol. 2017;168:26–37. Epub 2017/01/25. doi: 10.1016/j.jsbmb.2017.01.010 . 28115235

[51] Kuroishi T. Regulation of immunological and inflammatory functions by biotin. Can J Physiol Pharmacol. 2015;93(12):1091–6. Epub 2015/07/15. doi: 10.1139/cjpp-2014-0460 . 26168302

[52] Sullivan MR, McGowen K, Liu Q, Akusobi C, Young DC, Mayfield JA, et al. Biotin-dependent cell envelope remodelling is required for Mycobacterium abscessus survival in lung infection. Nat Microbiol. 2023;8(3):481–97. Epub 2023/01/20. doi: 10.1038/s41564-022-01307-5 . 36658396 PMC9992005

[53] Tanaka M, Yanagi M, Shirota K, Une Y, Nomura Y, Masaoka T, et al. Eosinophil and foam cell accumulation in lungs of Sprague-Dawley rats fed purified, biotin-deficient diets. Vet Pathol. 1995;32(5):498–503. Epub 1995/09/01. doi: 10.1177/030098589503200507 . 8578640

[54] Yan F, Wen Z, Wang R, Luo W, Du Y, Wang W, et al. Identification of the lipid biomarkers from plasma in idiopathic pulmonary fibrosis by Lipidomics. BMC Pulm Med. 2017;17(1):174. Epub 2017/12/08. doi: 10.1186/s12890-017-0513-4 . 29212488 PMC5719761

[55] Qin X, Lin X, Liu L, Li Y, Li X, Deng Z, et al. Macrophage-derived exosomes mediate silica-induced pulmonary fibrosis by activating fibroblast in an endoplasmic reticulum stress-dependent manner. J Cell Mol Med. 2021;25(9):4466–77. Epub 2021/04/10. doi: 10.1111/jcmm.16524 . 33834616 PMC8093963

[56] Magro CM, Allen J, Pope-Harman A, Waldman WJ, Moh P, Rothrauff S, et al. The role of microvascular injury in the evolution of idiopathic pulmonary fibrosis. Am J Clin Pathol. 2003;119(4):556–67. Epub 2003/04/25. doi: 10.1309/0B06-Y93E-GE6T-Q36Y . 12710128

[57] Xia Y, Cheng M, Hu Y, Li M, Shen L, Ji X, et al. Combined transcriptomic and lipidomic analysis of D-4F ameliorating bleomycin-induced pulmonary fibrosis. Ann Transl Med. 2021;9(18):1424. Epub 2021/11/05. doi: 10.21037/atm-21-3777 . 34733976 PMC8506780

[58] Marsland BJ, Trompette A, Gollwitzer ES. The Gut-Lung Axis in Respiratory Disease. Ann Am Thorac Soc. 2015;12 Suppl 2:S150–6. Epub 2015/11/26. doi: 10.1513/AnnalsATS.201503-133AW . 26595731

[59] Hübner RH, Gitter W, El Mokhtari NE, Mathiak M, Both M, Bolte H, et al. Standardized quantification of pulmonary fibrosis in histological samples. Biotechniques. 2008;44(4):507–11, 14–7. Epub 2008/05/15. doi: 10.2144/000112729 . 18476815

