# Supplementary figures and images for: The role of the microbiota and metabolites in the treatment of pulmonary fibrosis with UC-MSCs: Integrating fecal metabolomics and 16S rDNA analysis
Source: PLoS One. 2025 Jan 9;20(1):e0313989. doi: 10.1371/journal.pone.0313989 (PMC11717254; doi:10.1371/journal.pone.0313989)

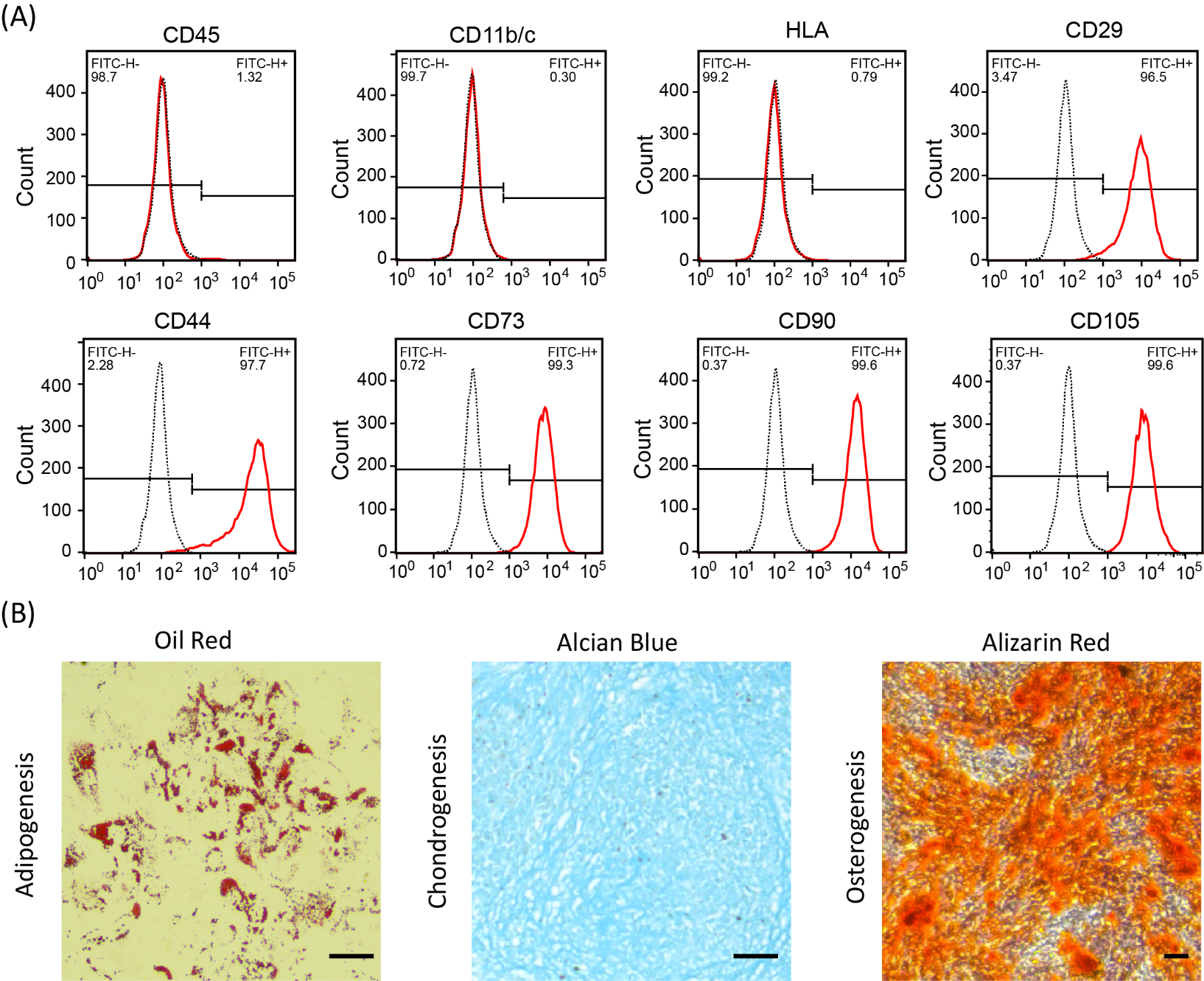

Supplement: S1 Fig — (A) Cells were analyzed by flow cytometry with the following antibodies: anti-CD11b/c, anti-CD45, anti-HLA-DR, anti-CD29, anti-CD44, anti-CD73, anti-CD90, and anti-CD105. (B) Identification of the potential of UC-MSCs for lipogenic, chondrogenic and osteogenic differentiation. (TIF) [file pone.0313989.s001.tif]

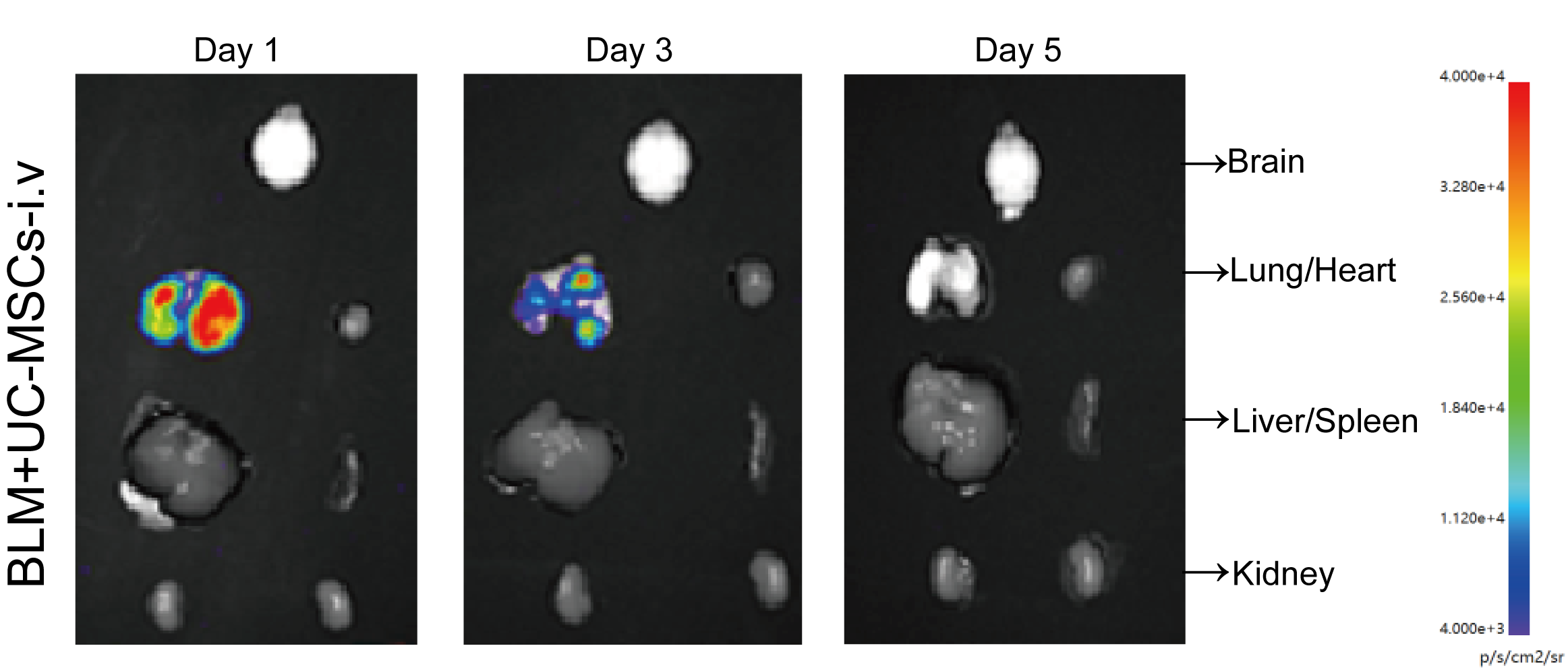

Supplement: S2 Fig — Fluorescence imaging of different ex vivo organs (brain, lung, heart, liver, spleen and kidney) 24 h after the administration of UC-MSCs. (TIF) [file pone.0313989.s002.tif]

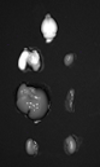

Supplement: S1 Data — (ZIP) [file pone.0313989.s003.zip › Day 5.tif]

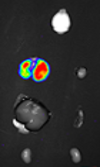

Supplement: S1 Data — (ZIP) [file pone.0313989.s003.zip › Day 1.tif]

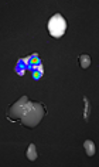

Supplement: S1 Data — (ZIP) [file pone.0313989.s003.zip › Day 3.tif]

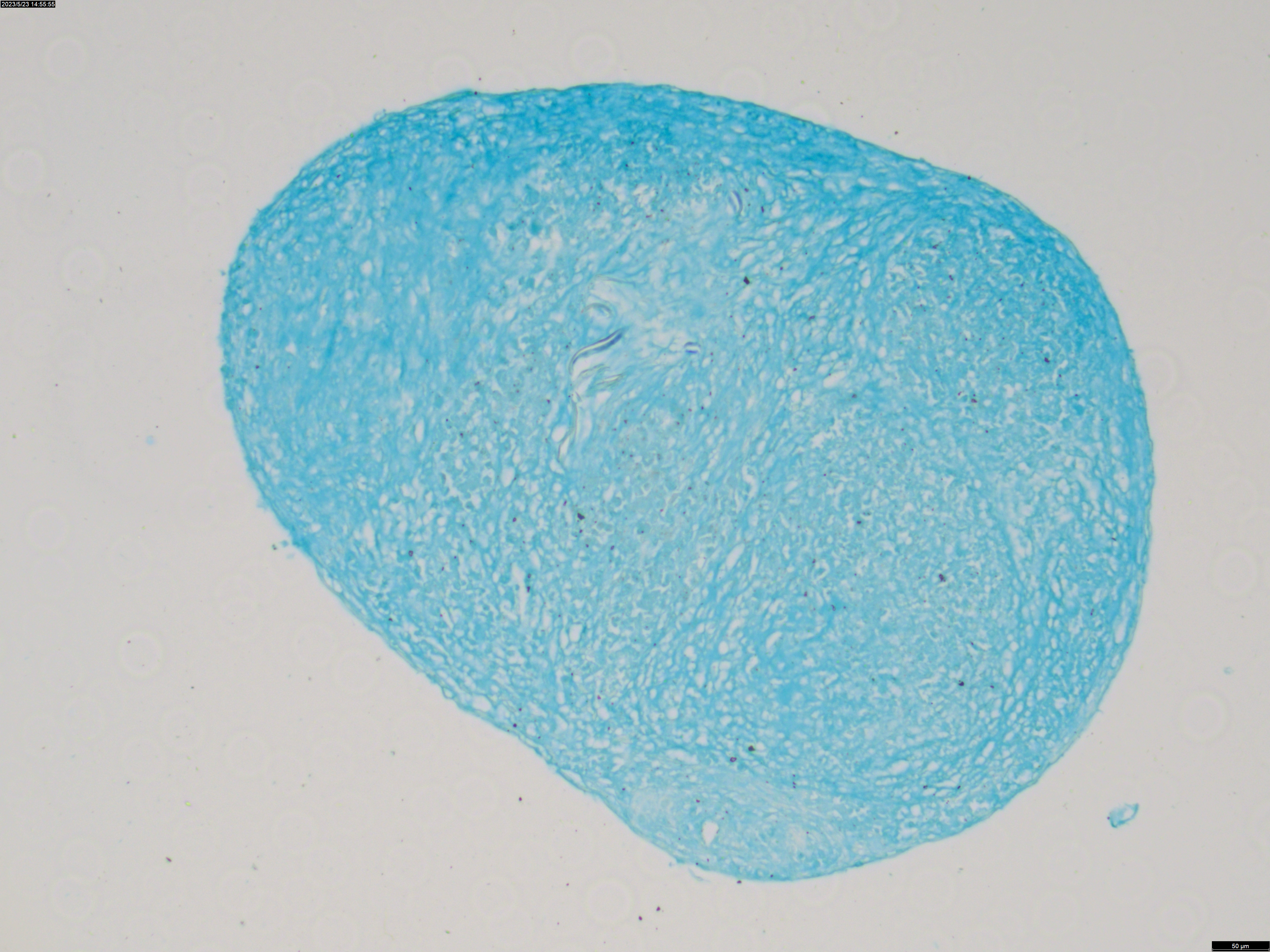

Supplement: S1 Data — (ZIP) [file pone.0313989.s003.zip › renamed_026fb.tif]

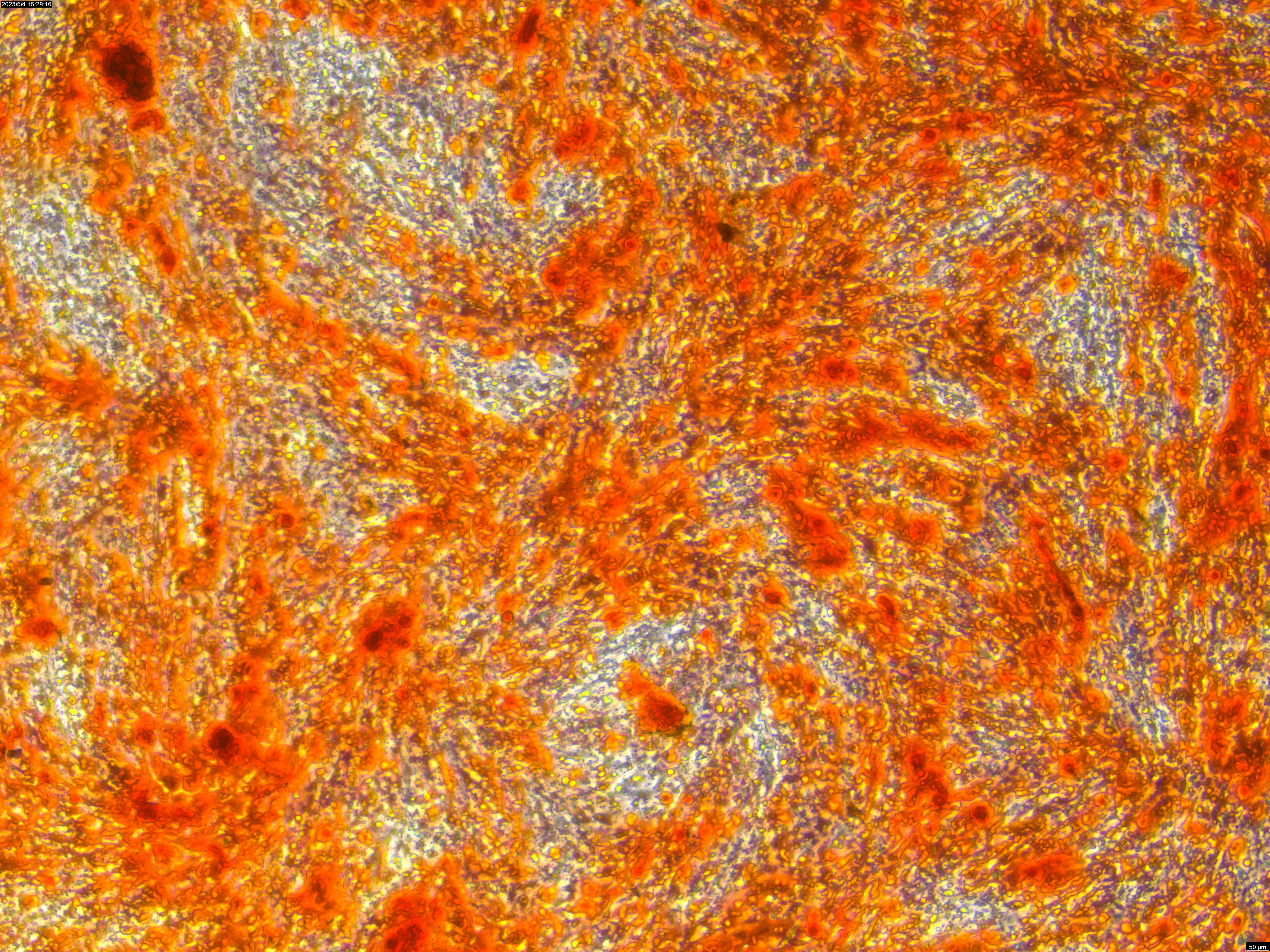

Supplement: S1 Data — (ZIP) [file pone.0313989.s003.zip › renamed_1dfc5.tif]

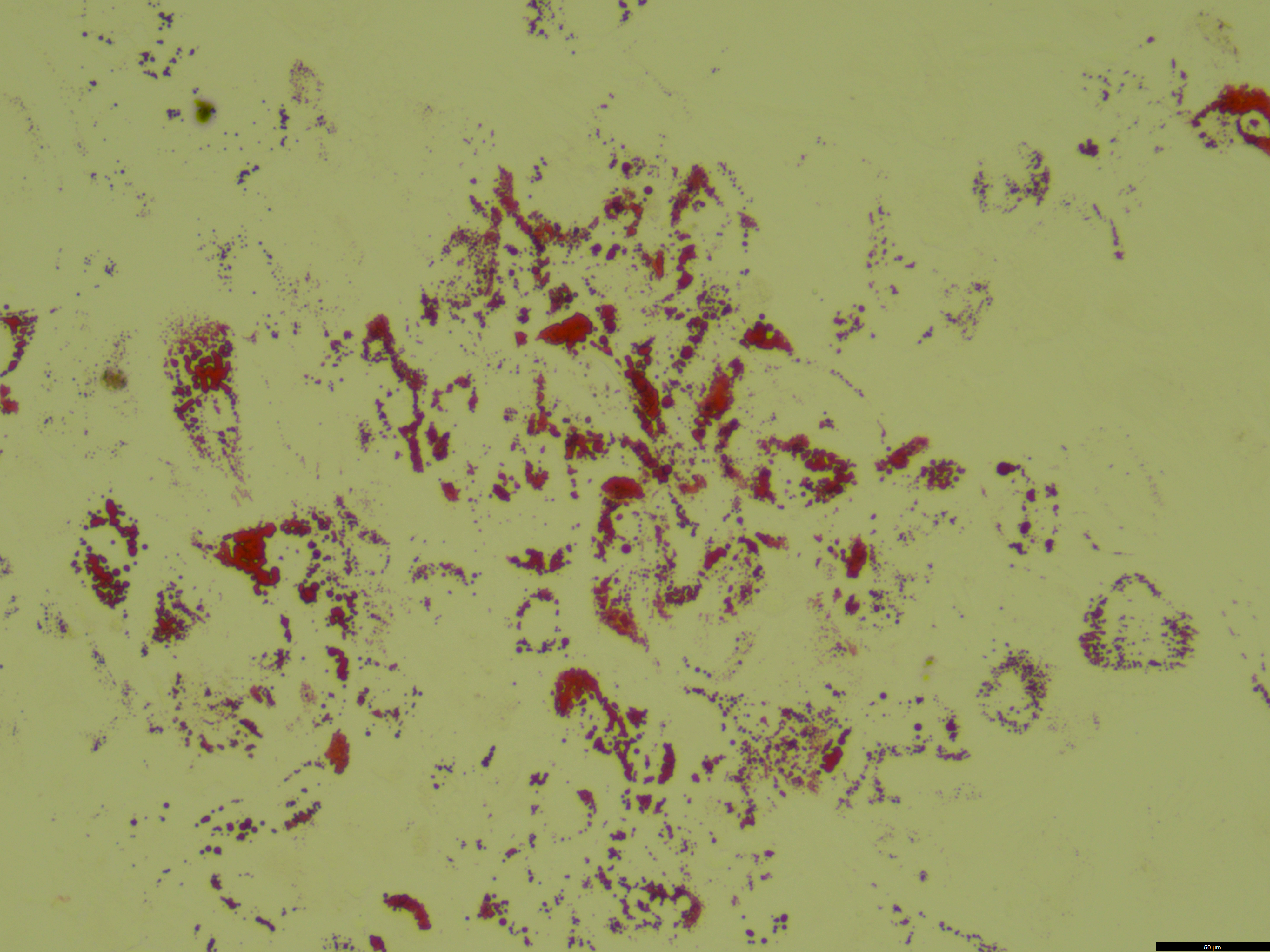

Supplement: S1 Data — (ZIP) [file pone.0313989.s003.zip › renamed_3c1e8.tif]
